# DNA–Protein Binding is Dominated by Short Anchoring Elements

**DOI:** 10.1002/advs.202414823

**Published:** 2025-03-26

**Authors:** Hong Chen, Yongping Xu, Hao Ge, Xiao‐Dong Su

**Affiliations:** ^1^ State Key Laboratory of Gene Function and Modulation Research, School of Life Sciences, and Biomedical Pioneering Innovation Center (BIOPIC) Peking University Beijing 100871 China; ^2^ Beijing International Center for Mathematical Research (BICMR) and Biomedical Pioneering Innovation Center (BIOPIC) Peking University Beijing 100871 China

**Keywords:** anchoring element (AE), anchoring element density (AED), transcription factor and DNA interaction, transcription factor binding site (TFBS)

## Abstract

Unveiling the complexities of gene expression regulation, the study explores the intricate DNA‐binding mechanisms of transcription factors (TFs). By employing the KaScape method previously developed to measure both bound and unbound populations at thermodynamic equilibrium, “anchoring elements” (AEs), 3–4 base pair sequences, are identified in *Arabidopsis* WRKY and human PU.1 TFs crucial for binding affinity. Building on the BEESEM method, the study introduces the AEEscape algorithm, which advances the AE concept by creating a precise model of the position‐specific k‐mer binding energy landscape. This method allows for the direct identification of the dominant role of AEs from experimental data. Moreover, when integrated with genomic data, it reveals an energetic funnel around transcription factor binding sites (TFBSs), which is directly correlated with the density of AEs (AED). The findings not only offer a fresh perspective on TF‐TFBS interactions but also highlight the critical role of AED in gene regulation. These insights can pave the way for innovative strategies to manipulate gene expression.

## Graphical abstract

This study delineate the binding mechanism of transcription factors (TFs) in the genome with the knowledge obtained at thermodynamic equilibrium conditions. Through KaScape experiments, combined with the AEEscape algorithm development, they identified the minimal dominant interacting elements, termed as Anchoring Elements (AEs). Their findings also demonstrate that AEs and AE density are important for TF binding and genome targeting.

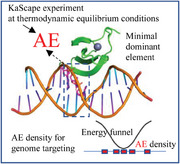

## 1 Introduction

Gene expression is central to cell development, growth and proliferation, with transcriptional regulation serving as a critical control point.^[^ ^1^ ^]^ Transcription factors (TFs) play a key role in this process, acting as conductors that bind to specific double‐stranded DNA (dsDNA) sequences known as transcription factor binding sites (TFBSs) to regulate gene transcription.^[^ ^2^, ^3^, ^4^ ^]^ Despite their fundamental importance, accurately identifying TFBSs remains a significant scientific challenge.^[^ ^5^, ^6^ ^]^

Traditionally, TFBSs are characterized by DNA binding motifs, often represented by position weight matrix (PWM), which quantify the probability of nucleotide presence at each position within the binding site.^[^ ^6^, ^7^, ^8^, ^9^ ^]^ Our comprehension of these motifs has been advanced by various experimental techniques, such as HT‐SELEX (high‐throughput systematic evolution of ligands by exponential enrichment),^[^ ^10^ ^]^ PBM (protein binding microarray),^[^ ^11^ ^]^ MITOMI (mechanically induced trapping of molecular interactions),^[^ ^12^ ^]^ and KaScape,^[^ ^13^ ^]^ along with in vivo techniques like ChIP (chromatin immunoprecipitation)‐chip,^[^ ^14^ ^]^ ChIP‐seq,^[^ ^15^ ^]^ as well as their modern variants.^[^ ^16^, ^17^, ^18^ ^]^ Computational models have also evolved to predict or directly identify TFBSs from dsDNA sequences, most of which are based on PWM.^[^ ^6^, ^19^, ^20^, ^21^, ^22^, ^23^ ^]^ However, these models have limitations due to the inherent degeneracy of TFBSs, which can lead to false positives and the overlooking of functionally relevant sites.^[^ ^21^, ^24^ ^]^ To address these challenges, higher‐order models considering nucleotide interdependencies have been developed, yet most of them focus primarily on dinucleotide interactions and often neglect the structural context of the DNA‐TF complex, which is essential for a comprehensive understanding of TF‐DNA interactions.^[^ ^25^, ^26^, ^27^, ^28^, ^29^ ^]^

The recognition of TFBSs by TFs is a critical and complex process in gene regulation.^[^ ^30^ ^]^ The prevailing hypothesis, “Facilitated Diffusion”, suggests that TFs locate their targets more efficiently than would be expected from random diffusion alone.^[^ ^31^, ^32^, ^33^, ^34^ ^]^ This theory posits that the genomic context surrounding TFBSs, particularly gene regulatory regions with repetitive degenerate sequences, plays a key role in guiding TFs during their search for binding sites.^[^ ^35^, ^36^, ^37^, ^38^, ^39^ ^]^ Moreover, an energy funnel surrounding TFBSs, potentially linked to base gradients, is believed to enhance the recognition of these sites.^[^ ^40^ ^]^

Recent research has underscore the role of short tandem repeats (STRs)—2‐6 bases repeats found widely in eukaryotic genomes—in modulating the local concentration of TFs, suggesting that STRs have a regulatory function in gene expression.^[^ ^41^ ^]^ However, the conventional motif‐based models of “specific” binding struggle to explain this phenomena. Insights from molecular dynamic simulation study has revealed that certain trinucleotide and tetranucleotide structures, as well as their energy profiles at transcription start sites, suggest the presence of a distinct signature profile for these elements.^[^ ^42^ ^]^ These findings highlight the need for further investigation into how eukaryotic TFs navigate and bind to their targets amid short sequence variations.

In this study, we introduce a new concept: “anchoring elements” (AEs), which are short dsDNA sequences of 3–4 base pairs crucial for TF binding. Our discovery, originated from KaScape experiments,^[^ ^13^ ^]^—where we analyzed the pull‐down dsDNA sequences population and eliminated input random sequence bias—has led to the development of the Anchoring Element Energy Landscape (AEEscape) algorithm. This algorithm performs a position‐specific m‐mer energy landscape analysis, enabling the identification of the minimal dsDNA sequence necessary for protein binding. The energy landscape generated by AEEscape reveals an energetic funnel around genomic binding peaks, where the AE density (AED) is essential for the rapid and precise targeting by TFs. This work advances our comprehension of TF‐DNA interactions and provides a new framework for exploring the complex mechanisms of gene regulation.

## 2 Results

### 2.1 Discovery of AE

To investigate the molecular interactions between TF and dsDNA, we first used the N‐terminal DNA binding domain (DBD) of *Arabidopsis thaliana* WRKY1 TF (*At*WRKY1N) as a representative model. Our experiment was based on the original KaScape method,^[^ ^13^ ^]^ with modifications to improve its effectiveness (Experimental Section/Methods 4.1; Figure S1, Supporting Information). KaScape is an in vitro method that comprehensively assesses the binding affinity of all possible k‐mers with a certain TF under thermodynamic equilibrium conditions, thereby enabling systematic study of TF‐DNA binding characteristics. Accordingly, we conducted a series of KaScape experiments, which primarily included pull‐down operations and sequencing with the length of the random base region set to 3, 4, and 5 base pairs. The input dsDNA pool and the TF‐bound dsDNA were sequenced, and their distributions were calculated, denoted as P(S_i_) and P(TS_i_|B), respectively. The input pool was comprehensive and highly random, ensuring minimal bias in the k‐mer sequence counts.^[^ ^13^ ^]^ To further reduce the input bias, we employed the relative binding energy, as defined in the KaScape method paper, using the input distribution as a divisor (Equation 1). To better approximate thermodynamic equilibrium, we modified the KaScape experiment by omitting the wash step and sequencing the DNA pool that did not bind to the protein, represented as P(S_i_|U). In this case, the relative binding energy was redefined (Equation 2), which aligns more closely with the definition of the association constant (Equations S1 and S2, Supporting Information).

(1) $$\mathrm{relative}\;\mathrm{binding}\;\mathrm{energy}=-\mathrm{lo}g_{2}\frac{P(TS_{i}\left|B\right)}{P(S_{i})}$$

(2) $$\mathrm{relative}\;\mathrm{binding}\;\mathrm{energy}=-\mathrm{lo}g_{2}\frac{P(TS_{i}\left|B\right)}{P(S_{i}\left|U\right)}$$

**Figure 1:**
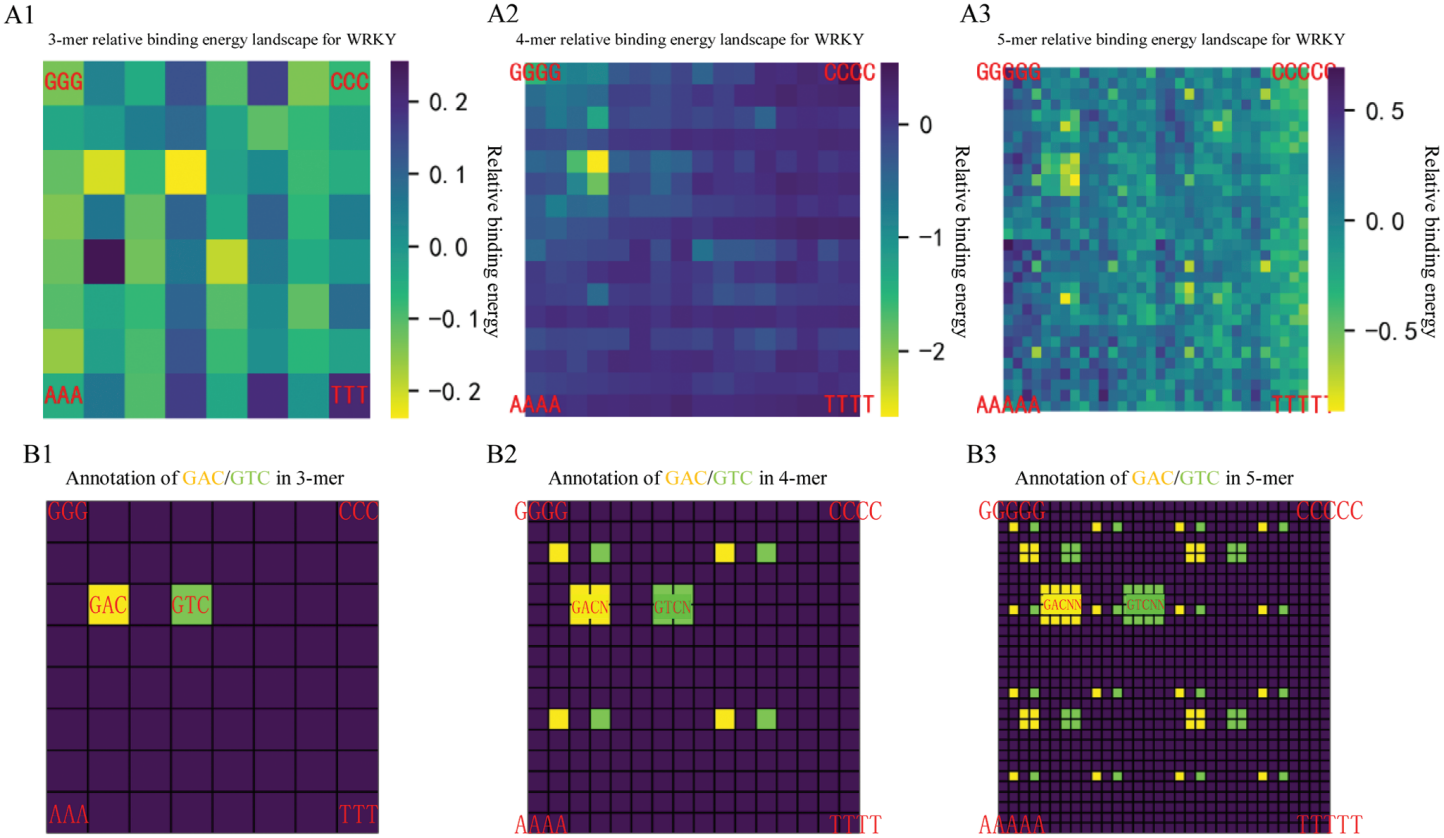
The KaScape relative binding energy landscapes on K‐mer graphs and the ideal model for *At*WRKY1N. A1–A3) The relative binding energy landscapes calculated directly from KaScape data. The random sequence type for each KaScape experiment is R1N3, R1N4 (the same with the data in reference),^[^ ^13^ ^]^ and R1N5, respectively. The data in Figure 1A3 result from a modified KaScape experiment, utilizing sequencing data obtained from both bound and unbound DNA libraries. B1–B3) The positions containing GAC and GTC are marked in yellow and green respectively for 3‐mer, 4‐mer, and 5‐mer graphs.

We introduced a color‐coded k‐mer graph (Figure S2, Supporting Information) mapping technique to characterize the relative binding energy landscapes derived from KaScape experimental datasets. In this representation, dark purple indicates high energy, while yellow represents low energy, which corresponds to higher binding affinity. The parameter “k” in the k‐mer graph map refers to the length of the random base sequence segment used in the KaScape experiment. The resulting binding energy landscape maps (**Figure** **1**A1–A3) displayed fractal‐like patterns, where a fractal is defined as a geometric structure that exhibits self‐similarity across different scales, with each subregion mirroring the complexity of the whole.

These fractal‐like patterns are attributed to sequences with lower binding energy that contain a certain short sequence of nucleotides, namely GAC or GTC‐tagged strings (GAC and GTC are reverse complement).^[^ ^43^ ^]^ The ideal fractal model is constructed using the k‐mer maps highlighting the presence of GAC and its reverse complement GTC sequences (Figure 1B1–B3). A comparative analysis between experimental data (Figure 1A1–A3) and the ideal model (Figure 1B1–B3) demonstrated a consistent pattern of high‐affinity binding site. These high‐affinity binding sites consistently incorporated the minimal elements, GAC or GTC. We thus have termed these shortest binding elements as Anchoring Elements (AEs) for their ability to bind the DBD protein long enough during the pull‐down experiments. The identification of AEs is fundamental, offering a molecular rationale for the observed binding specificity and affinity of DBD to its target dsDNA sequences.

### 2.2 The AEEscape Algorithm for AE Confirmation

**Figure 2:**
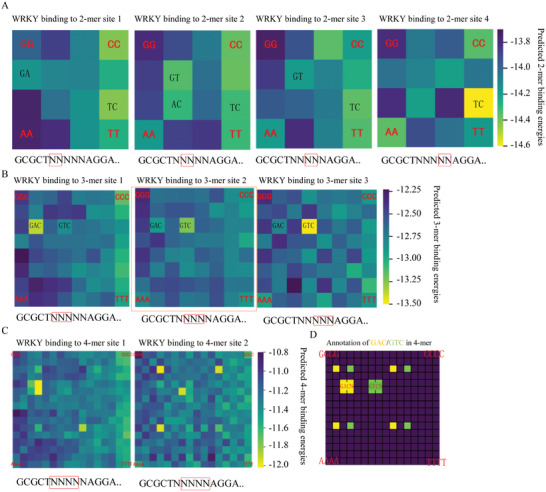
The binding energy landscapes at different locations in the random region predicted by the AEEscape algorithm trained using KaScape experimental data for *At*WRKY1N. The binding energy parameters in each row are simultaneously predicted by training using one KaScape experiment. The experimental dataset used for each row is the same as that used in Figure 1A3. The random sequence at the bottom of each figure is the sequence type used in the KaScape experiment (see Text S1, Supporting Information, for more detail). The red box in the random region at the bottom of the figure denotes the location of the predicted binding energy landscape. This allows for convenient visualization of the binding affinity of k‐mers at the exact location within the random region. The binding energy in the orange box is used to calculate the binding energy surrounding the genomic binding peaks. N represents G, C, A, or T. A) predicted 2‐mer binding energy landscapes at locations 1, 2, 3, and 4 in the random region with low binding energy marked in black. B) predicted 3‐mer binding energy landscapes at locations 1, 2, and 3 in the random region, with positions containing GAC and GTC labeled in black. C) predicted 4‐mer binding energy landscapes at locations 1, and 2 in the random region. D) The positions containing GAC and GTC are highlighted in yellow and green, respectively, in the 4‐mer graph. The annotation makes the specific sequence information of high‐affinity binding signals in the k‐mer graph more conspicuous. It allows for an at‐a‐glance understanding of the precise sequence details associated with high affinity, facilitating further summarization of the characteristics of transcription factor binding to DNA.

To further investigate the binding energy of short DNA sequences and confirm the presence of AEs, we developed the AEEscape algorithm. This computational approach calculates binding energies for m‐mer sequences across the random base region, as illustrated in **Figure** **2** for *At*WRKY1N. When the sequence length (m) was set to 2, the binding energy landscapes varied across different locations (Figure 2A; Figure S3, Supporting Information), with sequences TC, GA, AC, and GT exhibiting relatively lower binding energies. Upon incrementing m to 3, the binding energy landscape displayed a significant degree of uniformity (Figure 2B), suggesting a convergence in binding preferences at this sequence length. Notably, the 3‐mer sequence GAC (or its reverse complement GTC) consistently presented the lowest binding energy across all tested locations (Figure S4, Supporting Information). Specifically, at binding site 1, GAC exhibited the lowest binding energy, while at binding site 2, GTC demonstrated the lowest binding energy with GAC also showing relatively low energy. At binding site 3, GTC again displayed the lowest binding energy. The consistent low binding energy of both GAC and GTC at binding site 2 is particularly noteworthy. These observations suggest that *At*WRKY1N exhibits a preferential binding affinity for the 3‐mer sequence GAC (or GTC) over other 3‐mer sequences, independent of its location within the random base region. Further analysis with m set to 4 revealed that the sequences with the lowest binding energies at each location all contained the GAC (or GTC) (Figure 2C). For instance, at the first location, sequences GACT, GACC, and TGAC exhibited the lowest binding energies (Figure S5, Supporting Information, left panel), whereas at the second location, sequences GGTC, AGTC, CGTC, and GTCA were identified with high affinity (Figure S5, Supporting Information, right panel). The 2‐mers with relatively low binding energies (TC, GA, AC, GT) are subsets of the 3‐mer with low binding energy (GAC or GTC, reverse complement), and all 4‐mers with low binding energies (GACT, GACC, TGAC, GGTC, AGTC, CGTC, and GTCA) contain the 3‐mer with the lowest binding energy (GAC or GTC, reverse complement). Our thorough examination of the binding energies for 2‐mers, 3‐mers, and 4‐mers across the random base region, along with the analysis of inclusion relationships, indicates that the 3‐mer sequence (AE) is the minimal element essential for *At*WRKY1N to bind with dsDNA in a specific manner and to exert a dominant influence on binding.

To ascertain the precision and interpretability of our computational predictions, we conducted a comparative analysis between the experimentally determined binding probability ratios for *At*WRKY1N, denoted as P(TS_i_|B)/P(S_i_|U) (Figure S6D, Supporting Information), and the predictions generated by the AEEscape algorithm (as described in Equation (13), and visualized in Figure S6A–C, Supporting Information), parameterized with 2‐mer, 3‐mer, and 4‐mer sequence lengths. An increment in the hyperparameter of sequence length was associated with an increase in the predictive accuracy of the AEEscape algorithm, as depicted in Figure S7 (Supporting Information). Specifically, at a sequence length of 3, the Pearson correlation coefficient rapidly reached to 0.91, and the least squares error was significantly reduced. At this sequence length, the AEEscape algorithm's predictions closely matched the experimental data (Figure S6B,D, Supporting Information), effectively capturing the majority of the experimental observations and providing substantial validation for the essential role of minimal element AEs in DNA‐protein binding (For example, the binding of *At*WRKY1N to DNA is dominated by GAC or GTC).

In summary, the AEEscape algorithm discerningly identifies minimal, high‐affinity short sequence that exhibits consistent presence and uniqueness across all positions within random regions, confirming their role as AEs. This algorithm not only verifies the existence of AEs but also demonstrates their crucial contribution to the specificity of DBD‐dsDNA binding, providing a robust framework for understanding the complex interactions between TFs and DNA.

### 2.3 Universality of AEs in DBD Binding

**Figure 3:**
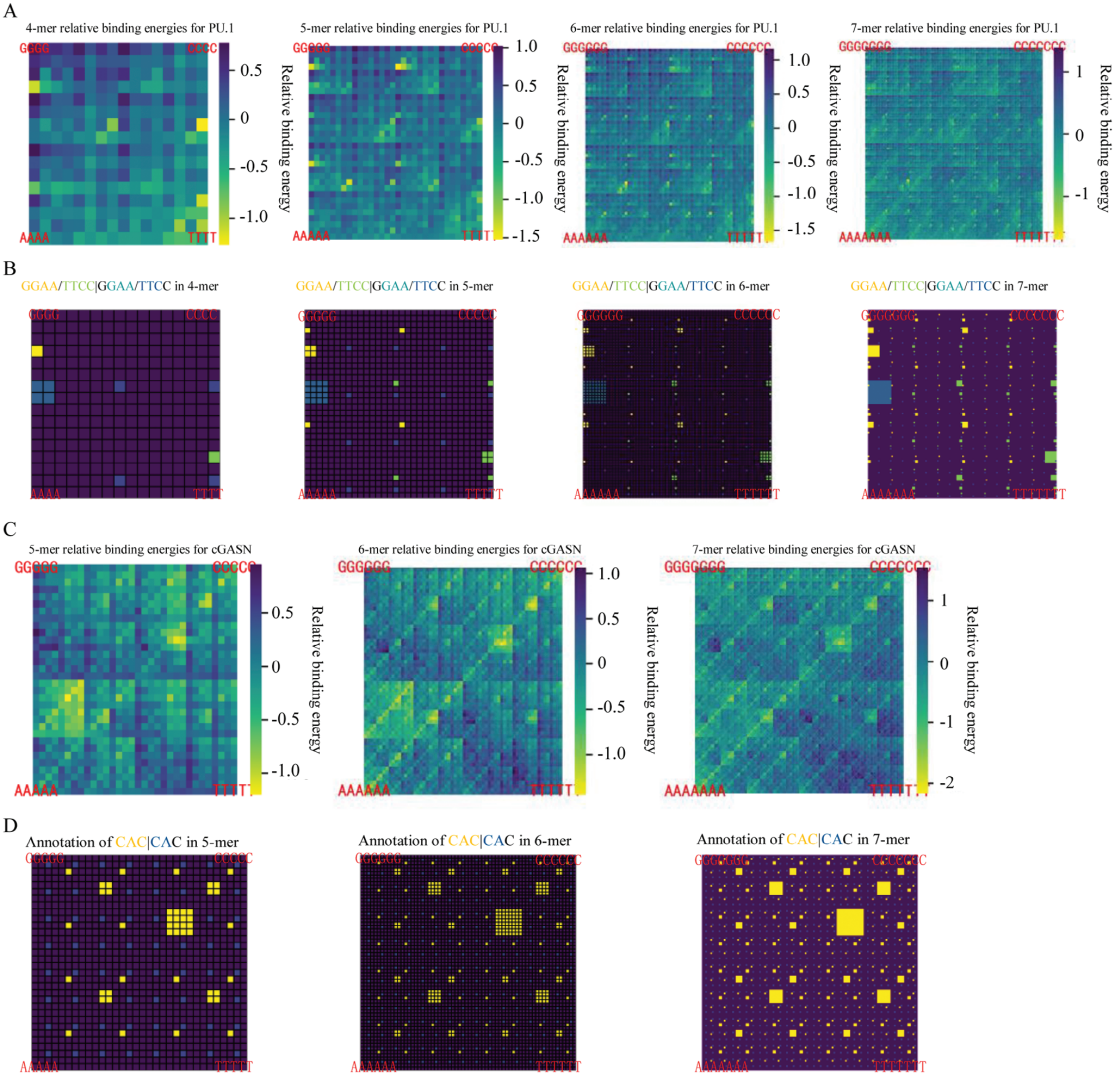
A series of KaScape relative binding energy landscapes on a K‐mer graph along with the ideal model for different TF DBDs. A) A series of KaScape experiments for PU.1 DBD. The random sequence type is R2N4, R2N5, R2N6, and R2N7. B) The positions containing GGAA and TTCC are highlighted in distinct colors for 4‐mer, 5‐mer, 6‐mer, and 7‐mer graphs. C) The KaScape experiment for cGASN. The random sequence type is R2N5, R2N6, and R2N7. D) The positions containing CAC are highlighted for 5‐mer, 6‐mer, and 7‐mer graphs.

To assess the prevalence of AEs across diverse DBDs, we performed a series of KaScape assays with a few very different DBDs, including the human PU.1 DBD, a representative of the ETS family of TFs, and the N‐terminal DBD of cyclic GMP‐AMP synthase (cGASN), belonging to a family of intrinsically disordered DBDs with an unidentified binding motif. Our analysis of the PU.1 DBD, leveraging KaScape and a series of random sequence libraries with random region length varying from 4 to 7 nucleotides, consistently revealed fractal‐like patterns (**Figure** **3**A). These patterns suggest the existence of a conserved minimal element crucial for binding, similar to what we found with *At*WRKY1N. A comparative analysis of the fractal‐like relative binding energy landscape map (Figure 3A) with the ideal fractal model (emphasizing GGAA and TTCC in the k‐mer map; Figure 3B) demonstrated that high‐affinity binding sequences invariably contained the AE GGAA or TTCC (the reverse complement). Parallel experiments with cGASN, using random sequence libraries with random region lengths of 5, 6, or 7, further validated the universality of AEs, with the fractal‐like patterns in the relative binding energy landscape maps being attributed to the AE CAC in sequences with low binding energy sequences (as compared in Figure 3C,D).

The AEEscape algorithm, trained on KaScape data, was subsequently used to compute the binding energy landscapes at different locations in the R2N7 random region for PU.1 DBD and cGASN. For PU.1 DBD, GGAA (or TTCC) consistently presented the lowest or second‐lowest relative binding energy across all locations (Figures S8A and S9, Supporting Information), solidifying its identification as the AE for PU.1 DBD. Similarly, for cGASN, the CAC consistently demonstrated the lowest relative binding energy (Figures S8B and S10, Supporting Information) across all locations, confirming its role as the AE for cGASN.

The AEs identified by AEEscape were found in agreement with those directly observed in the fractal‐like k‐mer graphs, validating their authenticity and prevalence. Figure S11 (Supporting Information) illustrates a comparison of the experimentally derived binding probability ratios, denoted as P(TS_i_|B)/P(S_i_) with those predicted by AEEscape, exhibiting a significant correlation. The Pearson correlation coefficients, calculated for PU.1 and cGASN, are 0.917 and 0.891, respectively. These coefficients provide robust evidence for the accuracy and interpretability of our computational predictions, thereby further validating the real existence and significance of AEs in mediating DBD‐dsDNA binding.

To ascertain whether the AE could be discerned independently from other experimental approaches, we analyzed PBM data for WRKY and PU.1.^[^ ^11^ ^]^ We computed the mean median intensity for 3‐mers, 4‐mers, 5‐mers, and 6‐mers, with the intensity for shorter sequences being the mean median signal intensity of 8‐mers encompassing the identical shorter sequence. Consistent with the KaScape experimental results, a fractal‐like pattern emerged (Figures S12 and S13, Supporting Information). Sequences with high intensity invariably contained the minimal short element or AE. Specifically, for WRKY, sequences containing GAC or GTC exhibited high signal intensity, while for PU.1, sequences with GGAA or TTCC displayed high signal intensity. These independent PBM experiments corroborated the existence of AE.

To further explore the general prevalence of AE, we analyzed the PBM data for a wide range of proteins, including ETS domain protein EHF, Fork head domain protein FOXJ3, GATA domain protein GATA3, Homeobox domain protein HOXB8 and PBX1, Homeobox, POU domain protein POU3f2, HLH domain protein MAX, HMG_box domain protein SOX4, T‐box domain protein TBX5, and NRF1.^[^ ^11^ ^]^ By calculating the normalized mean intensity for k‐mers, we again observed the fractal‐like phenomenon. The AE for each protein were determined and are highlighted in red, as detailed in Figures S14 and S15 and Table S1 (Supporting Information).

### 2.4 AE Density Gradient Facilitates TF Search to the Target

**Figure 4:**
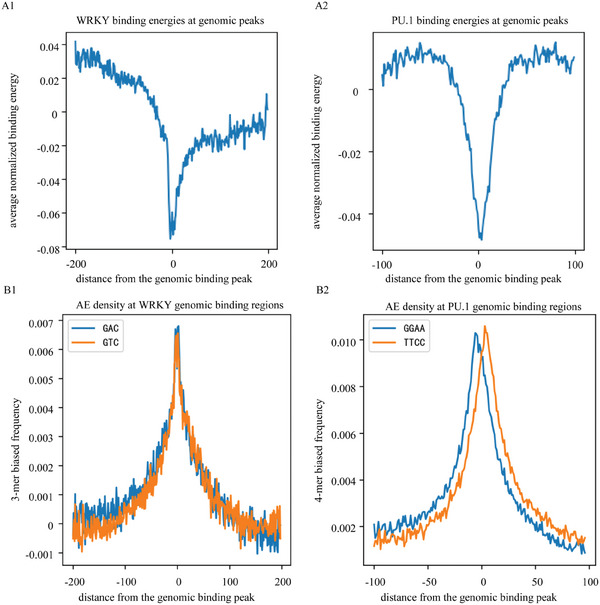
The average normalized binding energy and the average AE frequency bias near the genomic binding peak. The x axis represents the distance from the genomic binding peak. The origin on the x‐axis (x = 0) is designated to represent the position of the binding peak. The binding energy depicted in the figures is determined through the AEEscape algorithm. The energy values utilized for these calculations correspond to those indicated within the orange boxes in Figure 2 and Figure S8 (Supporting Information), respectively. A1) Binding energy around the genomic binding peak for WRKY. (A2) Binding energy around the genomic binding peak for PU.1. B1) The average frequency bias of AE (GAC or GTC for WRKY) in the vicinity of the genomic binding peak for WRKY. B2) The average frequency bias of AE (GGAA or TTCC for PU.1) in the vicinity of the genomic binding peak for PU.1.

We used the minimal binding energy profile, which plays a major role in TF binding, to explore the genomic search mechanisms of TFs. To identify potential functional sites for TFs, we localized genomic TFBS by analyzing the ReMap2022 dataset,^[^ ^44^ ^]^ which includes curated ChIP‐seq, ChIP‐exo, and DAP‐seq data.^[^ ^45^ ^]^ Binding peak locations in this dataset were considered as TFBS. We extracted genomic sequences adjacent to the TFBS (target sequence), excluding those with ambiguous nucleotides (N), and aligned them relative to the TFBS (Table S2, Supporting Information). The precise mapping of binding energies surrounding the annotated TFBS for WRKY and PU.1 TFs were facilitated by the AEEscape algorithm, as depicted within the orange box (m‐mer binding energy landscape) in Figure 2B and Figure S8A (Supporting Information). This algorithm, trained with KaScape experimental data, provided minimal essential binding energy profiles that represent the potential binding strength of these TFs to their target dsDNA sequences. Employing m‐mer binding energies, we scanned each target sequence using a sliding window approach (1 base pair stride) to generate the energy profile for the target sequence. We then used a distance‐dependent function (Equations 3 and 4) adapted from previous study,^[^ ^40^ ^]^ to calculate the normalized mean binding energy around TFBS. In Equation 3, $x_{k}^{\alpha }$ represents the position on the DNA of the kth target site of the TF. $E(x_{k}^{\alpha })$ denotes the energy of the k‐mer at position $x_{k}^{\alpha }$, which is generated by the AEEscape algorithm trained with the corresponding TF KaScape experimental data. The terms mean($E(x_{k}^{\alpha })$) and $\mathrm{std}(E(x_{k}^{\alpha }))$ indicate the mean and standard deviation of the k‐mer energy profile for each sequence, respectively. $E(x_{k}^{\alpha }+r)$ refers to the specific k‐mer energy at a distance r from the target. Finally, the normalized energy at distance r from the target are averaged over all targeted sequences. The resulting energy profiles revealed an energy gradient, or “funnel”, around the TFBS for both WRKY and PU.1 TFs, as visualized in **Figure** **4**A1,A2, which may assist in guiding the TF toward their target.

We subsequently sought to elucidate the mechanism underlying the energy funnel phenomenon. Since the energy funnel is influenced by the m‐mer composition surrounding the TFBS, and given that AE is the predominant m‐mer sequence, we analyzed AE enrichment near the TFBS. Specifically, we calculated the average AE frequency bias b(r) using Equations (5) and (6).^[^ ^40^ ^]^ This bias measures how much the AE frequency at a distance r from the TFBS deviates from the genome‐wide average. The indicator function I(x) in Equation (5) identifies the presence of the m‐mer sequence at position x. Figure 4B1,B2 illustrates the average AE frequency bias for WRKY and PU.1, respectively, showing a symmetrical, gradually increasing peak around the TFBS. This pattern differs from a simple delta function with a peak of 1 at the TFBS site and 0 elsewhere. For WRKY, the average frequency bias was calculated for all 3‐mers (Figure S16, Supporting Information), while for PU.1, we used 4‐mers (Figure S17, Supporting Information). The area under the peak curve represents the enriched k‐mer density, and we found that the AE density surrounding the ChIP‐seq peak region was the highest compared to other k‐mer sequences (GAC and GTC for WRKY; GGAA and TTCC for PU.1). This suggests a selective binding preference of the DBD for regions with high AED.

To examine whether this AED phenomenon is generalizable across TFs in their regulatory roles in vivo, we applied the same method to analyze the ReMap2022 data for a broad range of TFs, including EHF, FOXJ3, GATA3, HOXB8, PBX1, POU3f2, MAX, SOX4, TBX5, and NRF1. We calculated the average frequency bias for all k‐mers and presented these in k‐mer graphs (Figures S18 and S19, Supporting Information). For each protein, the average AE frequency bias was highlighted in a red box, exhibiting a symmetrical, progressively increasing peak that surpassed the peaks for other k‐mers. The area under the average frequency bias curve quantitatively reflects the density of each k‐mer. The area pattern in the k‐mer graph closely resemble those observed in the PBM k‐mer graphs (comparing Figures S14, S15, S18, and S19, Supporting Information). Larger areas corresponded to stronger intensity signals for the respective k‐mers, with AEs consistently occupying the largest area (highlighted in the red box). We further analyzed sequences that did not match the complete motif consensus, as obtained from the human TF^[^ ^10^ ^]^ and JASPAR databases. The symmetrical, gradually increasing peak for AEs was preserved for all proteins except HOXB8 (Figures S20 and S21, Supporting Information). In the case of HOXB8, the motif YMATTA closely resembles its AE, ATTA, leading to the exclusion of most sequences containing ATTA. However, the gradually increasing peak for ATAA, TAAA, TTAT, and TTTA remained, suggesting that these may represent secondary AEs for HOXB8, based on their relatively high PBM intensity (Figure S14, Supporting Information).

These findings provide a deeper understanding of how AEs influence TF binding specificity. They emphasize the crucial role of AED in facilitating the targeted binding of TFs to their regulatory sites in vivo.

(3) $$E(r)=\langle \frac{E\left(x_{k}^{\alpha }+r\right)-\mathrm{mean}\left(E\left(x_{k}^{\alpha }\right)\right)}{\mathrm{std}\left(E\left(x_{k}^{\alpha }\right)\right)}\rangle$$

(4) $$\;\langle Y\rangle =\frac{1}{N_{\mathrm{TF}}}\;\sum_{\alpha =1}^{N_{\mathrm{TF}}}\frac{1}{M_{\alpha }}\sum_{k=1}^{M_{\alpha }}Y_{k}^{\alpha }$$

(5) $$\beta \left(x\right)=I\left(x\right)-F_{\mathrm{element}}$$

(6) $$b(r)=\langle \beta \left(x_{k}^{\alpha }+r\right)\rangle$$

## 3 Discussion

**Figure 5:**
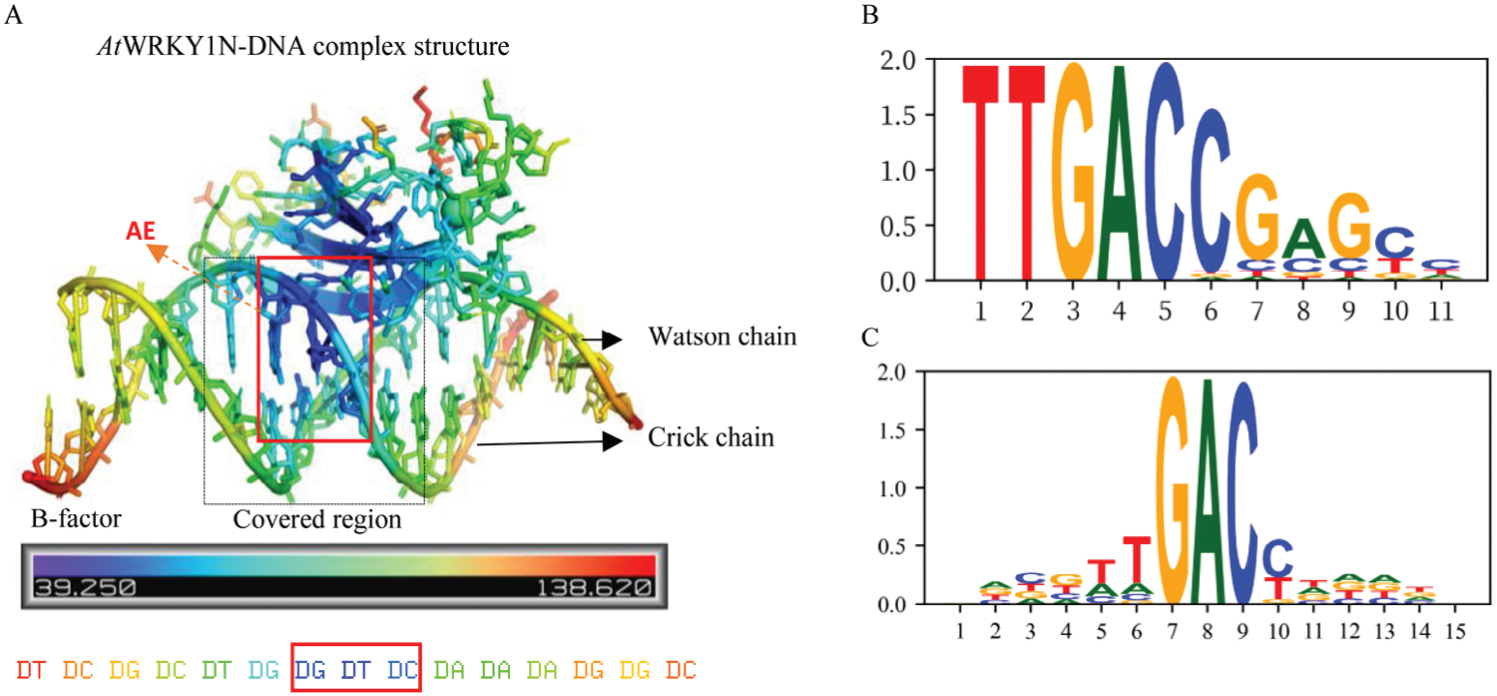
The structural and motif analysis of WRKY DBD‐DNA interaction. A) Complex structure of WRKY DBD with DNA (6j4e.pdb). The structure is colored according to B‐factor values, with bluer regions indicating lower B‐factor and greater stability. The corresponding DNA sequence of the Crick chain is displayed below, with the WRKY covered region highlighted in a black box and the AE marked in a red box. B) The PWM sequence logo from JASPAR database (MA0589.1). C) The PWM sequence logo derived from Uniprobe (UP00582).

In this study, we have refined the model of TF‐DNA interactions by focusing on the binding of monomeric DBDs to single binding sites. We conducted KaScape experiments using monomeric DBDs, such as *At*WRKY1N, PU.1 DBD, and cGASN, to investigate these interactions. Our experiments revealed fractal‐like, self‐correlated patterns in the binding affinity landscape. These patterns, driven by high‐affinity pull‐down sequences containing a shared short element (3‐4 bases), deviate from the traditional model, which proposes a single, high‐affinity binding consensus motif of longer sequences. This observation prompted the identification of a minimal binding element, which we term the “AE”. Building on this, we developed the AEEscape method, an extension of PWM analysis that calculates position‐specific m‐mer binding energies. This allows for the detection of genome‐wide energy gradients that correspond to TF binding preferences. We validated our method by analyzing independent PBM experimental data using K‐mer graph, which again revealed fractal‐like, self‐correlated patterns, confirming the universal presence of AEs (Figures S12–S15, Supporting Information). The AE represents a short, high‐information content sequence within the binding motif, aligning closely with current PWM models (Figure 5B; Figure S22B, Supporting Information). For example, in the sequence logo, “GAC” for WRKY and “GGAA” for PU.1 exhibit the highest information content. While PWM sequence logos can vary based on the experimental data and sequence alignments (Figure 5B,C), our KaScape‐based findings consistently and objectively identify the critical k‐mer (AE) that governs TF binding. Unlike PWM analysis, which assumes independent base interactions, our approach evaluates all possible k‐mers, providing a more comprehensive and accurate representation of binding preferences. Furthermore, the AEs identified in this study correspond to the “core sequences” commonly described in structural studies of DNA‐protein complexes. These core sequences—such as GTC for WRKY^[^ ^46^, ^47^ ^]^ and “GGAA” (or “TTCC”) for PU.1.^[^ ^48^ ^]^—represent the key bases that participate in electrostatic interactions and hydrogen bonding. These interactions are essential for the thermodynamic stability and “specificity” of binding, as reflected in their typically low “crystallographic B‐factors” (**Figure** **5**A; Figure S22A, Supporting Information). Like the AEs in our study, these “core sequences” are short and smaller than the overall motif lengths. Therefore, AEs can be viewed as the minimal structural units responsible for TF binding. This suggests that the short k‐mer sequence making up the AE interact with the TF simultaneously and should be considered as a whole, rather than as independent bases. The KaScape experimental enrichment results emphasize the importance of these structural “core sequences” in DNA binding mechanism and provide valuable insight into the role of these short sequences.

The AE, represented as the minimal building blocks of TF‐DNA interaction, is fundamentally determined by the TF's DBD protein fold. Generally, AEs can be used to classify TF domains, as members within the same TF domain (family) tend to share the same AE, whereas distinct TF domains are characterized by unique AEs. For instance, the AE for ETS domain TFs is commonly TTCC or GGAA.^[^ ^49^ ^]^ Within a given TF domain, binding specificity can vary due to differences in the flanking sequences adjacent to the AE. In eukaryotic systems, many TFs regulate cooperatively.^[^ ^50^ ^]^ The AEs for these TFs remain unchanged whether they are interacting with DNA alone or in combination with other TFs. As depicted in Figure S23 (Supporting Information), the AEs for each TF domain are highlighted within red boxes, which are identical when they interact with DNA alone. In these scenarios, the consideration of multiple AEs and their cooperative effects is necessary. AEs, as the fundamental unit of protein‐DNA binding, allow for a more detailed investigation of how protein domains cooperate in DNA binding. Although the affinity differences between AEs and other m‐mers are not substantial due to their short length, multivalent interactions from multiple nearby AEs can considerably enhance the overall binding affinity. This concept provides a basis for understanding STR‐TF binding.^[^ ^41^ ^]^ Furthermore, the AE content within a sequence provides an initial binding affinity, which can be further enhanced by alterations in the flanking sequence, as exemplified by the PU.1 TF, where the AE (core sequence) GGAA/TTCC alone is sufficient for binding at the micromolar (µM) range, the surrounding sequence variations may increase the binding affinity to nanomolar (nM) levels.^[^ ^48^ ^]^

By analyzing the genomic TF binding sequences from the ReMap2022 dataset, we identified regions with enriched AED within the TF binding regions (Figures S16–S19, Supporting Information). These enriched AEDs seem to facilitate the attraction of TFs, thereby maintaining a high local population of TF binding. Interestingly, the AED enrichment persisted even after excluding binding sequences containing complete consensus motif (Figures S20 and S21, Supporting Information). This finding challenges the traditional binary model of TF target recognition, which typically relies on identifying consensus motifs and searching for them within the genomic sequence to define TFBSs.^[^ ^51^, ^52^, ^53^, ^54^ ^]^ Our results suggest a co‐evolutionary relationship between DNA sequences and TFs. Specifically, regions with higher AED appear to show greater binding stability and faster evolutionary adaptation, which help secure and refine TF binding sites, in contrast to regions with lower AED. The robust mechanism of TF binding search allows for the accommodation of higher mutational loads, thanks to the collective density of AEs around the TFBS.^[^ ^55^ ^]^

This study focuses on the simplest scenario: monomeric TF binding to free dsDNA in the absence of histones. However, it lays the groundwork for more complex systems. Future research could expand on this approach and concept by investigating TFs that interact with DNA in dimeric forms or possess multiple functional domains, as well as those that require co‐factors or auxiliary proteins for their activity. Additionally, a deeper understanding of the interactions between nucleosomal DNA and pioneer factors could be better achieved. Extending this approach could provide valuable insights into gene regulation within the native chromatin context of eukaryotic genomes.^[^ ^56^, ^57^, ^58^, ^59^ ^]^

## 4 Experimental Section

### Experimental Data Generated Using the KaScape Method

The KaScape experiments, as previously reported,^[^ ^13^ ^]^ were conducted on the following DBDs: *At*WRKY1N, PU.1 DBD, and cGASN. These experiments generated sequencing reads from either a randomized dsDNA pool (input) or the corresponding DBD bound dsDNA (bound), constituting the input‐bound dataset. Text S1 (Supporting Information) details the random sequence types used. For *At*WRKY1N, the experiments utilized R1N3 and R1N4 sequences, where “R1” represents a constant flanking sequence and “N3” and “N4” denote the number of randomized bases. In contrast, the interactions with PU.1 DBD and cGASN employed R2N4, R2N5, R2N6, and R2N7 sequences, with “R2” signifying an alternative constant flanking sequence and “N4” to “N7” indicating an incremental increase in randomized bases (Table S3, Supporting Information).

To enhance the KaScape approach, modifications were introduced: The 5 × 10^−11^ mol protein was mixed with 5 × 10^−11^ mol dsDNA in a buffer solution composed of 25 mm HEPES, pH 7.0, 100 mm NaCl to a final volume of 200 µL, and the mixture was incubated on ice for 30 min, a typical condition in biochemical experiments. Subsequently, 1 µL of balanced magnetic beads was added to the mixture. The mixture was gently rotated at ≈10 rpm for 1 h at 4 °C to prevent protein denaturation. To minimize DNA‐protein dissociation and attain thermodynamic equilibrium, the wash step was omitted. Bound and unbound dsDNAs were then isolated and sequenced to generate the unbound‐bound dataset. The R1N5 sequence was exclusively used for *At*WRKY1N in the modified experiments (Table S3, Supporting Information). The input‐bound dataset was predominantly utilized for analysis, with exceptions noted in the text.

### AEEscape Biophysical Model

The AEEscape biophysical model, which is based on the BEESEM model,^[^ ^60^ ^]^ characterizes the binding energy of each m‐mer element at different locations in the random base region. In the BEESEM model, the binding ability of a specific m‐mer sequence s_j_ is characterized by calculating the ratio (denoted by R_j_) of P(s_j_|B) (the fraction of the bound sequences where the binding sequence is s_j_) and $\tilde{P}\left(s_{j}\right)$ (the fraction of s_j_ before binding to the TF) (Equation 7). P(B) is the binding probability of all types of dsDNA sequences; u is the chemical potential of the TF; and E(s_j_) is the binding energy of s_j_ regardless of its location on the sequence and is calculated using a PWM, which assumes that the bases in the motif are independent of each other. In the PWM model, changing one base in the motif does not affect the energy contribution of the nearby unchanged bases; however, there are correlations between bases when TFs are bound, which can provide insight into the binding mechanism. To explore the binding mechanism, we developed the AEEscape algorithm, hypothesizing a short binding site length (m) of 2 to 4 for the sequence s_j_ based on the AE concept derived from KaScape's k‐mer landscape analysis. Due to the influence of the constant flanking sequence, the binding energy landscape of s_j_ may be different at different locations in the random base region; therefore, $s_{j}^{k}$ was introduced to represent a specific m‐mer sequence at the kth location in the random bases. For simplicity, only TFs that were bound to random base regions were considered. The length of random bases in each sequence was denoted by l. There are l‐m+1 binding sites. $E_{j}^{k}$ is the binding energy between $s_{j}^{k}$ and TF. Equation (7) is generalized by replacing s_j_ with $s_{j}^{k}$ (Equation 8). Instead of using PWM to represent the energy as BEESEM does, we take $E_{j}^{k}$ directly as a parameter in AEEscape. Finally, we use the EM (Expectation‐Maximization) algorithm to compute $E_{j}^{k}$, the chemical potential, and the auxiliary parameters. The loss function is shown in Equation (9) (see Text S2, Supporting Information, for a detailed description).

(7) $$R_{j}=\frac{P\left(s_{j}|B\right)}{\tilde{P}\left(s_{j}\right)}=\frac{1}{P(B)}\;\frac{e^{-E(s_{j})}}{e^{-E(s_{j})}+e^{-u}}=R_{j\theta }\;$$

(8) $$R_{j}^{k}=\frac{P\left(s_{j}^{k}|B\right)}{\tilde{P}\left(s_{j}^{k}\right)}=\frac{1}{P(B)}\;\frac{e^{{-E}_{j}^{k}}}{e^{{-E}_{j}^{k}}+e^{-u}}{=R}_{j\theta }^{k}$$

(9) $$\mathrm{Error}\;=\sum_{\mathrm{jk}}{\left(R_{j}^{k}-R_{j\theta }^{k}\right)}^{2}$$

### Training and Evaluation of AEEscape

There are two types of KaScape datasets that are used to train the AEEscape model (described above): the input dsDNA P(S_i_) and the output dsDNA P(TS_i_|B) (input‐output dataset) as well as the unbound dsDNA P(S_i_|U) and the output dsDNA (unbound‐output dataset). For the second dataset, P(S_i_|U) and F_UB_ (the ratio of total sequencing reads in unbound versus bound) were used to represent the ratio of P(S_i_) and P(B) (Equation S25, Supporting Information) and rearrange Equation S20 (Supporting Information) to obtain $Q_{j}^{k}$ (Equation S26, Supporting Information), which is similar to $R_{j}^{k}$. The loss function is shown in Equation (10) (see Text S2, Supporting Information, for a detailed description).

The binding association constant for each long sequence S_i_ used in the KaScape experiment is denoted as Ka(S_i_) and is calculated using Equation (11). The $E_{j}^{k}$ is predicted using the AEEscape algorithm. Utilizing Equations (12) and (13), the ratios of the probabilities P(TS_i_|B) to P(S_i_), and P(TS_i_|B) to P(S_i_|U), respectively, were predicted. These ratios can also be directly ascertained from the experimental data. Thereby, the Pearson correlation coefficient and the least square error between the predicted values and those derived from the experimental data is employed to evaluate the performance of the algorithm.

(10) $$\mathrm{Error}\;=\sum_{jk}{\left(Q_{j}^{k}-Q_{\theta j}^{k}\right)}^{2}$$

(11) $$\mathrm{Ka}\;\left(S_{i}\right)=\sum_{k}I_{\mathrm{ij}}^{k}e^{{-E}_{j}^{k}}\;$$

(12) $$\;\frac{P(TS_{i}\left|B\right)}{P(S_{i})}=\frac{\sum_{k}I_{\mathrm{ij}}^{k}e^{{-E}_{j}^{k}}}{\left(\sum_{k}I_{\mathrm{ij}}^{k}e^{{-E}_{j}^{k}}+e^{-u}\right)P(B)}\;$$

(13) $$\frac{P(TS_{i}\left|B\right)}{P(S_{i}\left|U\right)}=\frac{F_{\mathrm{UB}}\sum_{k}I_{\mathrm{ij}}^{k}e^{{-E}_{j}^{k}}}{e^{-u}}$$

## Conflict of Interest

The authors declare no conflict of interest.

## Supporting information



Supporting Information

## Data Availability

The data that support the findings of this study are available from the corresponding author upon reasonable request.
