# Supplementary material for: DNA–Protein Binding is Dominated by Short Anchoring Elements
Source: Adv Sci (Weinh). 2025 Mar 26;12(19):2414823. doi: 10.1002/advs.202414823 (PMC12097035; doi:10.1002/advs.202414823)
Supplement: Supplementary file 1 — Supporting Information [file ADVS-12-2414823-s001.pdf]

ADVANCED SCIENCE
Open Access

Supporting Information

for *Adv. Sci.*, DOI 10.1002/advs.202414823

DNA–Protein Binding is Dominated by Short Anchoring Elements

*Hong Chen, Yongping Xu, Hao Ge and Xiao-Dong Su**

Supporting Information

DNA–protein binding is dominated by short Anchoring Elements

*Hong Chen, Yongping Xu, Hao Ge, and Xiao-Dong Su**

Supporting Information Text

1. randomized dsDNA sequences used in the text

Random sequence type 1:

R1N3: GCGCTNNNAGGAGTGGGATCCGGGGGGGG

R1N4: GCGCTNNNNAGGAGTGGGATCCGGGGGGGG

R1N5: GCGCTNNNNNAGGAGTGGGATCCGGGGGGGG

Random sequence type 2:

R2N4: ACTCAGTGNNNNCTAGTACGAGGAGATCTGCATCTC

R2N5: ACTCAGTGNNNNNCTAGTACGAGGAGATCTGCATCTC

R2N6: ACTCAGTGNNNNNNCTAGTACGAGGAGATCTGCATCTC

R2N7: ACTCAGTGNNNNNNNCTAGTACGAGGAGATCTGCATCTC

2.1 The biophysical model

This section describes the details of the AEEscape biophysical model in two parts. In the first part, the experimental data used in the model is an input-bound dataset, while in the second part, it is an unbound-bound dataset. And the second part is slightly adjusted based on the first part. The biophysical model is based on BEESEM[1].

We denote the random nucleotide length in the random dsDNA sequence by l. In the system, there are 4^l different sequences (denoted by S_i for each type of sequence). The transcription factor (TF) is denoted by T. We use $T{\cdot}S_i$ to represent the TF-dsDNA complex. X^U is used to represent the unbound X molecules. We assume that the system is in equilibrium (Equation S1). And the association constant for each type of sequence is Equation S2, where [X] is the concentration of molecule X.

$$T^U+S_i^U \rightleftharpoons T{\cdot}S_i \qquad \text{(S1)}$$

$$K_a=\frac{[T{\cdot}S_i]}{[T^U][S_i^U]} \qquad \text{(S2)}$$

2.2 The BEESEM algorithm

Given that the binding site (s_j) is shorter than the sequence read, multiple binding locations can be occupied by the transcription factor. The central tenet of the BEESEM model is to estimate the binding probability of the transcription factor to the binding site within the bound sequences, denoted as $P(Ts_j|B)$. There are two distinct methods to calculate this probability, as elaborated in Equation S7 and S11, respectively. By rearranging the formula presented in S7, we derive the ratio R_j and $R_{\theta j}$ given in Equation S10. In Equation S10, P(Tsj|B) can be replaced with the expression from S11. The least squares function, as outlined in Equation S12, serves as the loss function for the model. The binding energy (E_j) between the transcription factor and the binding site is characterized by the Position Weight Matrix (PWM) model, which is obtained by summing the binding energies of each base, under the assumption of independence among the bases. The model is trained using the Expectation-Maximization (EM) algorithm, which facilitates the computation of the PWM energies and other ancillary parameters.

$$P\left(Ts_j\right)=\sum_i \sum_{k=1}^{l\text{-}m+1} I_{ij}^k\, P\left(TS_i^k|S_i\right)P(S_i)=\sum_i \frac{\sum_{k=1}^{l\text{-}m+1} I_{ij}^k \left[TS_i^k\right]}{[S_i]} P(S_i) \qquad \text{(S3)}$$

$$=\sum_i \frac{\sum_{k=1}^{l\text{-}m+1} I_{ij}^k\, e^{-E_j}}{\sum_{q=1}^{l\text{-}m+1}\left[e^{-E_i^q}\right]+e^{-u}} P(S_i)$$

$$I_{ij}=\begin{cases}1, & \text{if } S_i^k=s_j\\ 0, & \text{if } S_i^k\neq s_j\end{cases} \qquad \text{(S4)}$$

$$E_j=-\ln K_{aj} \qquad \text{(S5)}$$

$$u=\ln\left[T^U\right] \qquad \text{(S6)}$$

$$P\left(Ts_j\middle|B\right)=\frac{P\left(Ts_j\right)}{P(B)}=\frac{\sum_i \frac{\sum_{k=1}^{l\text{-}m+1} I_{ij}^k\, e^{-E_j}}{\sum_{q=1}^{l\text{-}m+1}\left[e^{-E_i^q}\right]+e^{-u}} P(S_i)}{P(B)}=\frac{1}{P(B)}\frac{e^{-E_j}}{e^{-E_j}+e^{-u}}\sum_i \frac{e^{-E_j}+e^{-u}}{\sum_{q=1}^{l\text{-}m+1}\left[e^{-E_i^q}\right]+e^{-u}} I_{ij}P(S_i) \qquad \text{(S7)}$$

$$w_{ij}=\frac{e^{-E_j}+e^{-u}}{\sum_{q=1}^{l\text{-}m+1}\left[e^{-E_i^q}\right]+e^{-u}} \qquad \text{(S8)}$$

$$P\left(Ts_j\middle|B\right)=\frac{1}{P(B)}\frac{e^{-E_j}}{e^{-E_j}+e^{-u}}\sum_i w_{ij}I_{ij}P(S_i) \qquad \text{(S9)}$$

$$R_j=\frac{P\left(Ts_j\middle|B\right)}{\sum_i w_{ij}I_{ij}P(S_i)}=\frac{1}{P(B)}\frac{e^{-E_j}}{e^{-E_j}+e^{-u}}=R_{\theta j} \qquad \text{(S10)}$$

$$P\left(Ts_j\middle|B\right)=\sum_i \frac{\sum_{k=1}^{l-m+1} I_{ij}^k\, e^{-E_j}}{\sum_{q=1}^{l\text{-}m+1}\left[e^{-E_i^q}\right]} P(TS_i|B) \qquad \text{(S11)}$$

$$\text{Error}=\sum_j (R_j-R_{\theta j})^2 \qquad \text{(S12)}$$

2.3 AEEscape Algorithm: Extension of BEESEM Algorithm

The AEEscape algorithm is a derivative of the BEESEM algorithm. In BEESEM, the binding energy between transcription factors (TFs) and binding sites is determined using the PWM model. This model presupposes the independence of 1-mer bases. AEEscape extends this concept by considering not only 1-mers but also 2-mers, 3-mers, 4-mers, and longer k-mers. In the AEEscape algorithm, the conditional probability of TF binding, originally denoted as $P(Ts_j|B)$ in the BEESEM model, is redefined as $P(Ts_j^k|B)$. This new notation represents the binding probability of a specific k-mer sequence at the kth binding site s_j^k among all bound sequences. Here, Ts_j^k signifies the binding event involving the TF and the k-mer s_j^k, with K_{aj}^k and E_j^k representing the binding association constant and the binding energy, respectively (Equation S15). The chemical potential of the TF is represented by u (Equation S16). An indicator function I_{ij}^k is introduced to determine whether a sequence S_i^k matches s_j^k (Equation S14). Utilizing E_j^k, u, and the sequence proportion $P(S_i)$ derived from the input double-stranded DNA (dsDNA) experimental data, the binding probability of Ts_j^k can be ascertained (Equation S13). The term B encompasses all types of bound dsDNA sequences, with P(B) representing the collective binding probability of these sequences. The conditional probability $P(Ts_j^k|B)$ is derived from $P(S_i)$ through a series of equations (Equation S17, S18 and S19). Employing bound dsDNA data, denoted as $P(TS_i|B)$, provides an alternative perspective on $P(Ts_j^k|B)$ (Equation S21). By rearranging Equation S19, the objective function is formulated (Equation S20 and S22). The binding energy E_j^k for m-mers at the kth location, where m ranges from 2 to 4 and beyond, serves as a direct parameter in the model. The unknown parameters E_j^k, u, and P(B) are subsequently determined through an Expectation-Maximization (EM) algorithm that minimizes the error.

$$P\left(Ts_j^k\right)=\sum_i P\left(Ts_j^k|S_i\right)P(S_i)=\sum_i \frac{I_{ij}^k\left[Ts_j^k\right]}{[S_i]}P(S_i)=\sum_i \frac{I_{ij}^k\left[Ts_j^k\right]}{\sum_{q=1}^{l-m+1}[TS_i^q]+\left[S_i^U\right]}P(S_i) \tag{S13}$$

$$=\sum_i \frac{\frac{I_{ij}^k\left[Ts_j^k\right]}{[T^U]\left[S_i^U\right]}}{\frac{\sum_{q=1}^{l-m+1}[Ts_i^q]}{[T^U]\left[S_i^U\right]}+\frac{\left[S_i^U\right]}{[T^U]\left[S_i^U\right]}}P(S_i)=\sum_i \frac{I_{ij}^k K_{aj}^k}{\sum_{q=1}^{l-m+1}[K_{ai}^q]+\frac{1}{[T^U]}}P(S_i)=\sum_i \frac{I_{ij}^k e^{-E_j{}^k}}{\sum_{q=1}^{l-m+1}\left[e^{-E_i^q}\right]+e^{-u}}P(S_i)$$

$$I_{ij}^{k}=\begin{cases}1, & \text{if } S_i^k=s_j^k\\ 0, & \text{if } S_i^k\neq s_j^k\end{cases} \quad \text{(S14)}$$

$$E_j^k=-\ln K_{aj}^k \quad \text{(S15)}$$

$$u=\ln\left[T^U\right] \quad \text{(S16)}$$

$$P\left(Ts_j^k\middle|B\right)=\frac{P\left(Ts_j^k\right)}{P(B)}=\frac{\sum_i \frac{I_{ij}^k e^{-E_j^k}}{\sum_{q=1}^{l-m+1}\left[e^{-E_i^q}\right]+e^{-u}}P(S_i)}{P(B)}=\frac{1}{P(B)}\frac{e^{-E_j^k}}{e^{-E_j^k}+e^{-u}}\sum_i \frac{e^{-E_j^k}+e^{-u}}{\sum_{q=1}^{l-m+1}\left[e^{-E_i^q}\right]+e^{-u}}I_{ij}^k P(S_i)$$

(S17)

$$w_{ij}^k=\frac{e^{-E_j^k}+e^{-u}}{\sum_{q=1}^{l-m+1}\left[e^{-E_i^q}\right]+e^{-u}} \quad \text{(S18)}$$

$$P\left(Ts_j^k\middle|B\right)=\frac{1}{P(B)}\frac{e^{-E_j^k}}{e^{-E_j^k}+e^{-u}}\sum_i w_{ij}^k I_{ij}^k P(S_i) \quad \text{(S19)}$$

$$R_j^k=\frac{P\left(Ts_j^k\middle|B\right)}{\sum_i w_{ij}^k I_{ij}^k P(S_i)}=\frac{1}{P(B)}\frac{e^{-E_j^k}}{e^{-E_j^k}+e^{-u}}=R_{\theta j}^k \quad \text{(S20)}$$

$$P\left(Ts_j^k\middle|B\right)=\sum_i \frac{I_{ij}^k e^{-E_j^k}}{\sum_{q=1}^{l-m+1}\left[e^{-E_i^q}\right]}P(TS_i|B) \quad \text{(S21)}$$

$$\text{Error}=\sum_{jk}\left(R_j^k-R_{\theta j}^k\right)^2 \quad \text{(S22)}$$

For the second part, $P(S_i)$, which is needed in the first part, is unknown. However, it can be derived from the unbound dsDNA experimental data $P(S_i|U)$ (Equation S23). C_B and C_U are the total number of sequencing reads for the bound and unbound dsDNA, respectively. With the relationship of $P(B)$, C_B, and C_U (Equation S24), we can obtain the ratio of $P(S_i)$ and $P(B)$ (Equation S25). Here F_{UB} is the ratio of C_U and C_B. Rearranging Equation S19, we obtain Equation S26, which is similar to Equation S20. Using Equation S11 to replace $P\left(Ts_j^k\middle|B\right)$ and Equation S25 to replace $\frac{P(S_i)}{P(B)}$, the unknown parameters E_j^k, u and F_{UB} can be

calculated from experimental data $P(TS_i|B)$ and F_{UB}. Again, we use the same EM algorithm as in the first part (with the loss function denoted in Equation S27).

$$P(S_i)=\frac{C_B P(TS_i|B)+C_U P(S_i|U)}{\sum_i[C_B P(TS_i|B)+C_U P(S_i|U)]}=\frac{P(TS_i|B)+\frac{C_U}{C_B}P(S_i|U)}{\sum_i\left[P(TS_i|B)+\frac{C_U}{C_B}P(S_i|U)\right]}=\frac{P(TS_i|B)+\frac{C_U}{C_B}P(S_i|U)}{1+\frac{C_U}{C_B}} \quad \text{(S23)}$$

$$P(B)=\frac{C_B}{C_B+C_U}=\frac{1}{1+\frac{C_U}{C_B}} \quad \text{(S24)}$$

$$\frac{P(S_i)}{P(B)}=P(TS_i|B)+\frac{C_U}{C_B}P(S_i|U)=P(TS_i|B)+F_{UB}P(S_i|U) \quad \text{(S25)}$$

$$Q_j^k=\frac{P(Ts_j^k|B)}{\sum_i w_{ij}^k I_{ij}^k \frac{P(S_i)}{P(B)}}=\frac{e^{-E_j^k}}{e^{-E_j^k}+e^{-u}}=Q_{\theta j}^k \quad \text{(S26)}$$

$$\text{Error}=\sum_{jk}(Q_j^k-Q_{\theta j}^k)^2 \quad \text{(S27)}$$

Supporting Information Figures

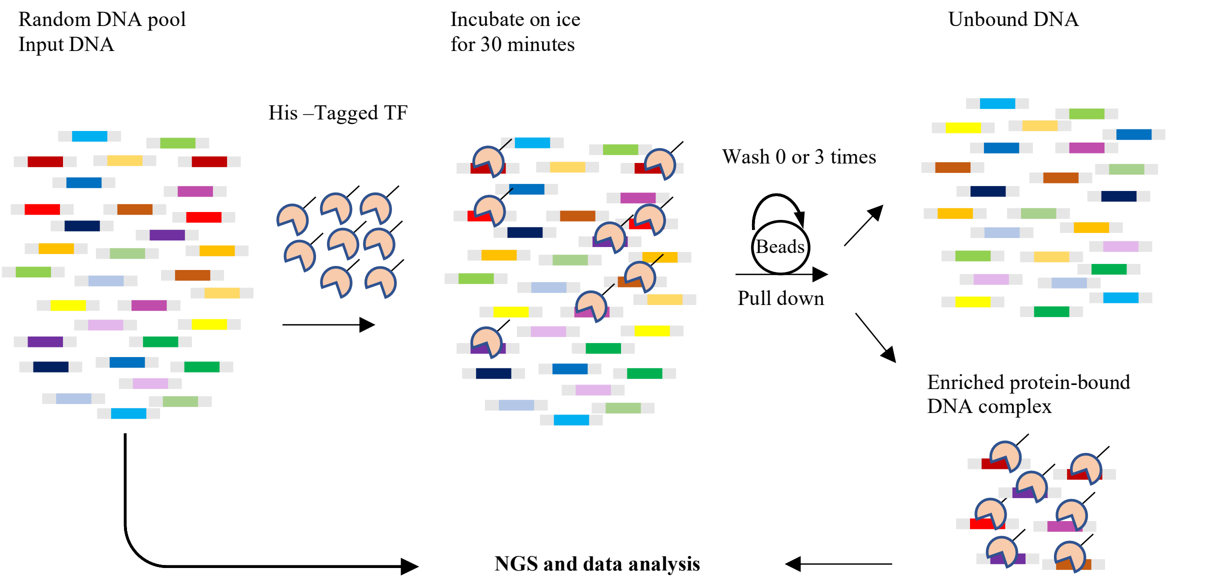

Figure S1. Schematic description of the KaScape process[2], not to scale. The random dsDNA pool (n = 4, 5, 6, or 7), represented by colored rectangular bars above, and the His-tagged TF DBD were prepared. The pooled dsDNAs consisted of approximately 30 base pairs with flanking sequences (Table S3). Next, 10^{-10}mol protein and 10^{-10}mol dsDNA were mixed in 2 mL buffer (or a one-tenth of the scale). The TF-DBD and dsDNAs were then incubated on ice for 30 min. Magnetic His-tag purification beads were added, and rotation was performed for one hour at 4°C, and the system was then washed and rotated 3 times. The dsDNA and TF-DBD complexes were then separated from the free unbound dsDNAs. Finally, the random dsDNA pool, unbound dsDNAs and bound dsDNAs were extended and used to produce the dsDNA library separately for next-generation sequencing.

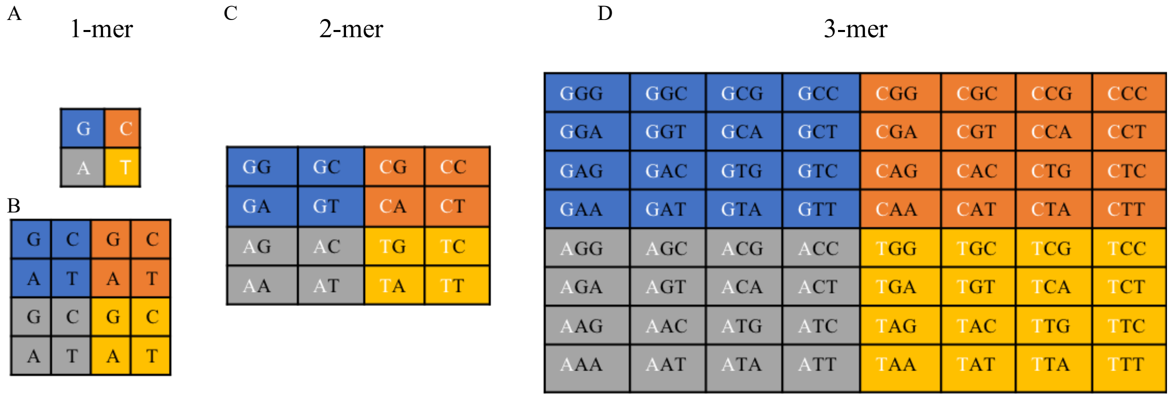

Figure S2. The K-mer graph. (A) 1-mer graph. (B) fourfold replication of 1-mer graph. (C) 2-mer graph. (D) 3-mer graph. The K-mer graph generation method from 1-mer to 2-mer can be extended to those longer than 2-mers. The generation of the 2-mer graph initiates with the duplication of a 1-mer graph (Figure S2A), resulting in four identical copies (Figure S2B). Subsequently, each short sequence is modified by the addition of a nucleotide base G, C, A, or T to the left side, yielding the 2-mer graph (Figure S2C). This method of iterative duplication and base extension is analogously applied for the synthesis of higher-order k-mer graphs.

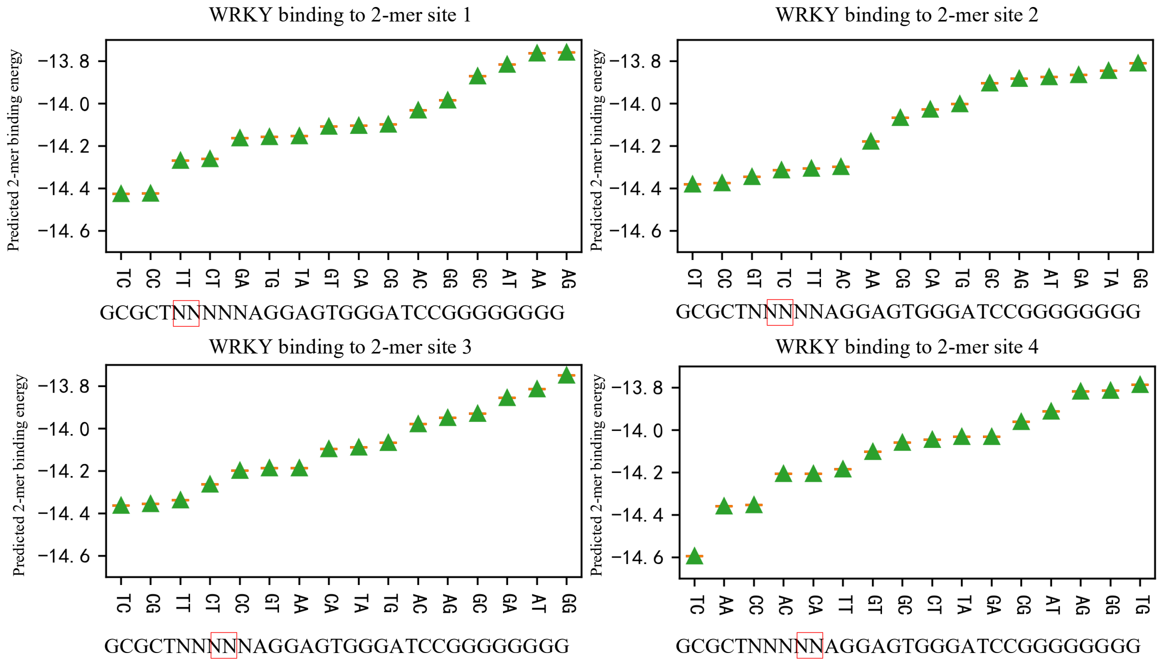

Figure S3. Plots of 2-mer binding energies at location 1, 2, 3, and 4 in the random region predicted by AEEscape for *At*WRKY1N. The red box in the random region at the bottom of the figure indicates the location of the predicted binding energy landscape. The binding energies are the same as in Figure 2A. The 32 2-mer sequences with the lowest binding energies are shown.

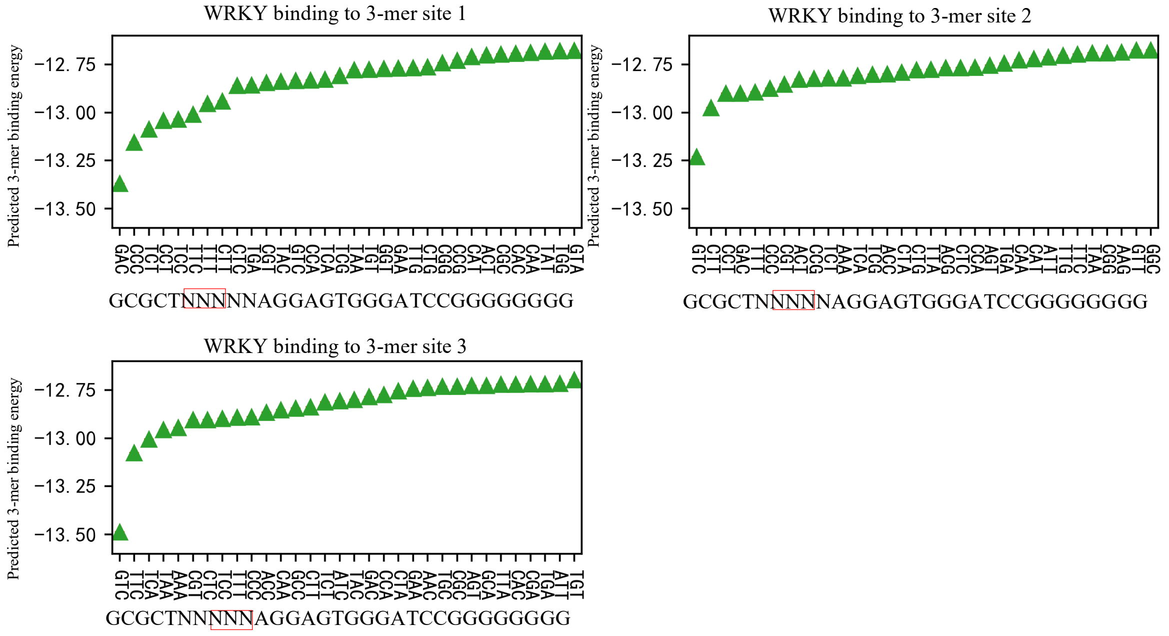

Figure S4. Plots of 3-mer binding energies at location 1, 2, and 3 in the random region predicted by AEEscape for *At*WRKY1N. The red box in the random region at the bottom of the figure indicates the location of the predicted binding energy landscape. The binding energies are the same as in Figure 2B. The 32 3-mer sequences with the lowest binding energies are shown.

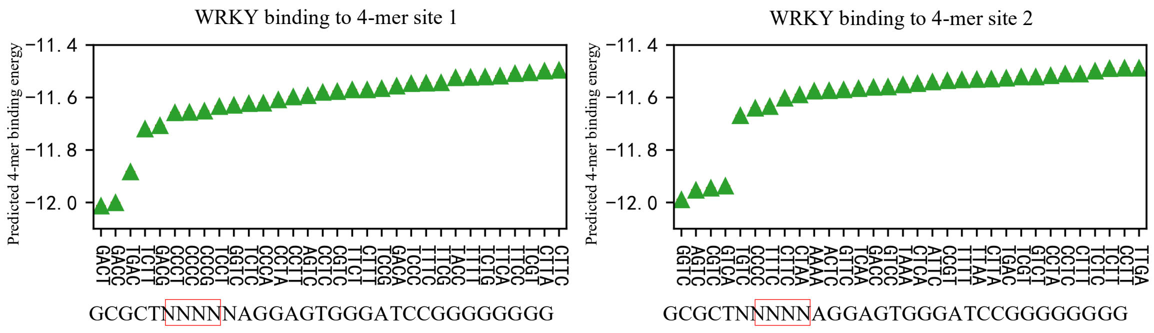

Figure S5. Plots of 4-mer binding energies at location 1 and 2 in the random region predicted by AEEscape for *At*WRKY1N. The red box in the random region at the bottom of the figure indicates the location of the predicted binding energy landscape. The binding energies are the same as in Figure 2C. The 32 4-mer sequences with the lowest binding energies are shown.

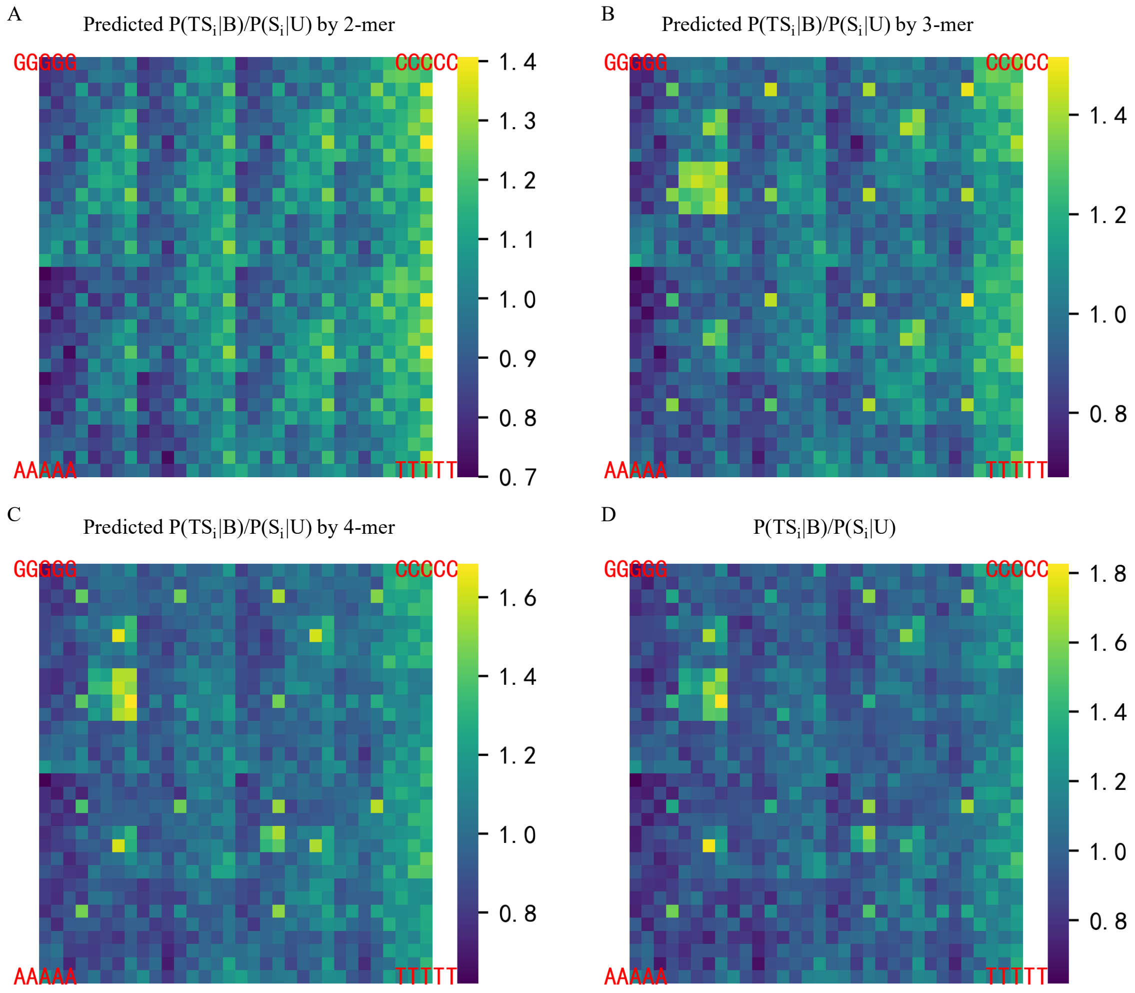

Figure S6. Comparative analysis of *At*WRKY1N binding probability ratios $P(TS_i|B)$ to $P(S_i|U)$, derived from AEEscape algorithm predictions with varying sequence length parameters and corroborated by KaScape experimental data. (A) The AEEscape algorithm, parameterized with a sequence length of 2, predicts the binding probability ratio, employing the binding energy parameters detailed in Figure 2A. (B) The AEEscape algorithm, parameterized with a sequence length of 3, predicts the binding probability ratio, employing the binding energy parameters detailed in Figure 2B. (C) The AEEscape algorithm, parameterized with a sequence length of 4, predicts the binding probability ratio, employing the binding energy parameters detailed in Figure 2C. (D) Directly observed binding probability ratios from KaScape experiments for *At*WRKY1N.

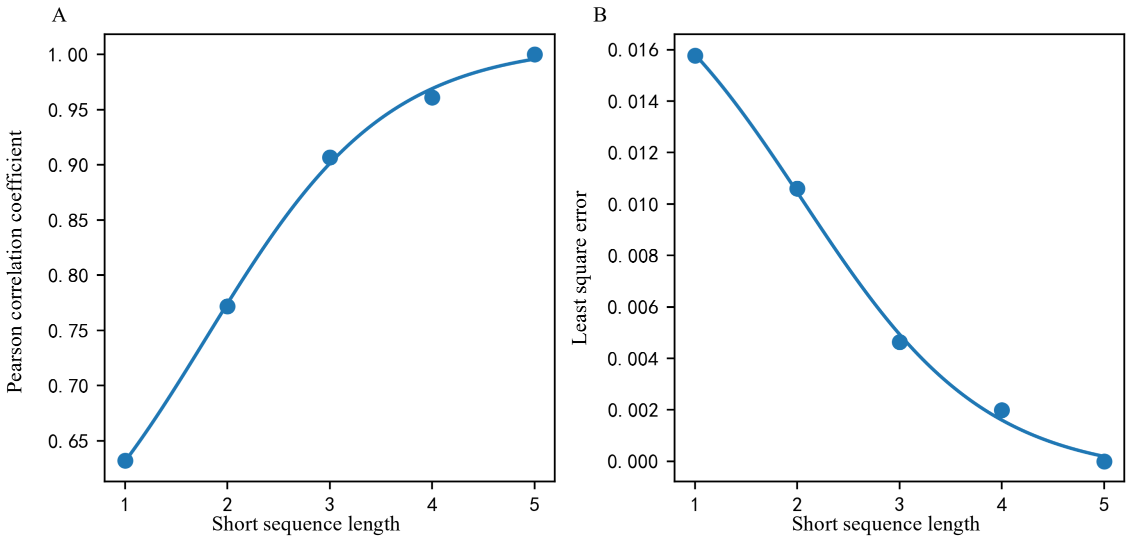

Figure S7. Accuracy assessment of the AEEscape algorithm. (A) Evaluation of the correlation between the binding probability ratios $P(TS_i|B)/P(S_i|U)$ derived from KaScape experiments for *At*WRKY1N and those predicted by the AEEscape algorithm across a series of short sequence length hyperparameters using the Pearson correlation coefficients. (B) Evaluation of the correlation between the binding probability ratios $P(TS_i|B)/P(S_i|U)$ derived from KaScape experiments for *At*WRKY1N and those predicted by the AEEscape algorithm across a series of short sequence length hyperparameters using the least square error.

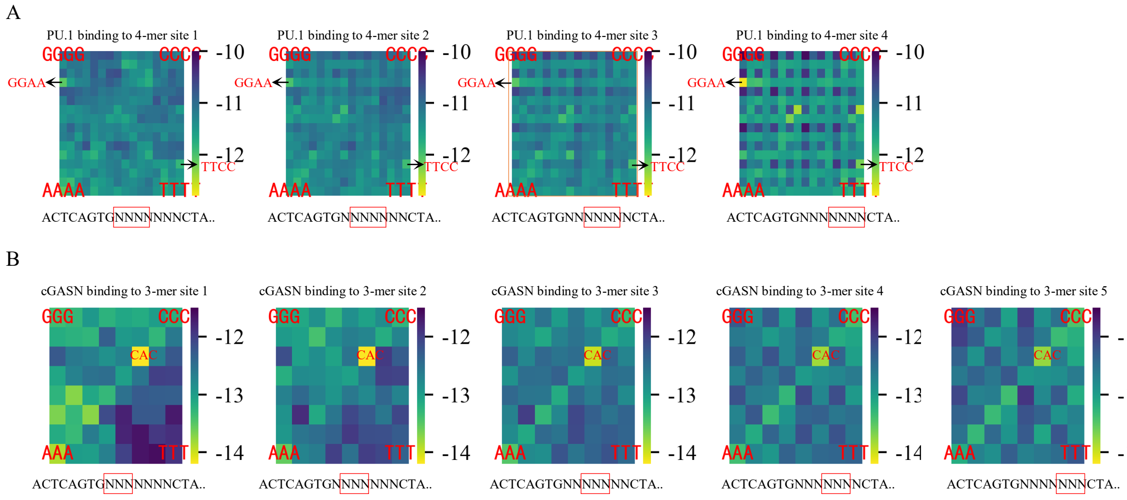

Figure S8. The binding energy landscapes at different locations in the random region predicted by the AEEscape algorithm. Each row represents simultaneous predictions from a single type of KaScape experiment for distinct proteins. The random sequence at the bottom denotes the sequence type used in the KaScape experiment. The red box in the random region

indicates the location of the predicted binding energy landscape. The binding energy in the orange box is used to calculate the binding energy near the transcription factor's genomic binding peaks. (A) Predicted 4-mer binding energy landscapes at locations 1, 2, 3, and 4 in the random region for PU.1. The KaScape experimental data used to predict are identical to those utilized in calculating the 7-mer relative binding energy for PU.1, illustrated in Figure 3A. (B) Predicted 3-mer binding energy landscapes at locations 1, 2, 3, 4, and 5 in the random region for cGASN. The KaScape experimental data used to predict are identical to those utilized in calculating the 7-mer relative binding energy for cGASN, illustrated in Figure 3C.

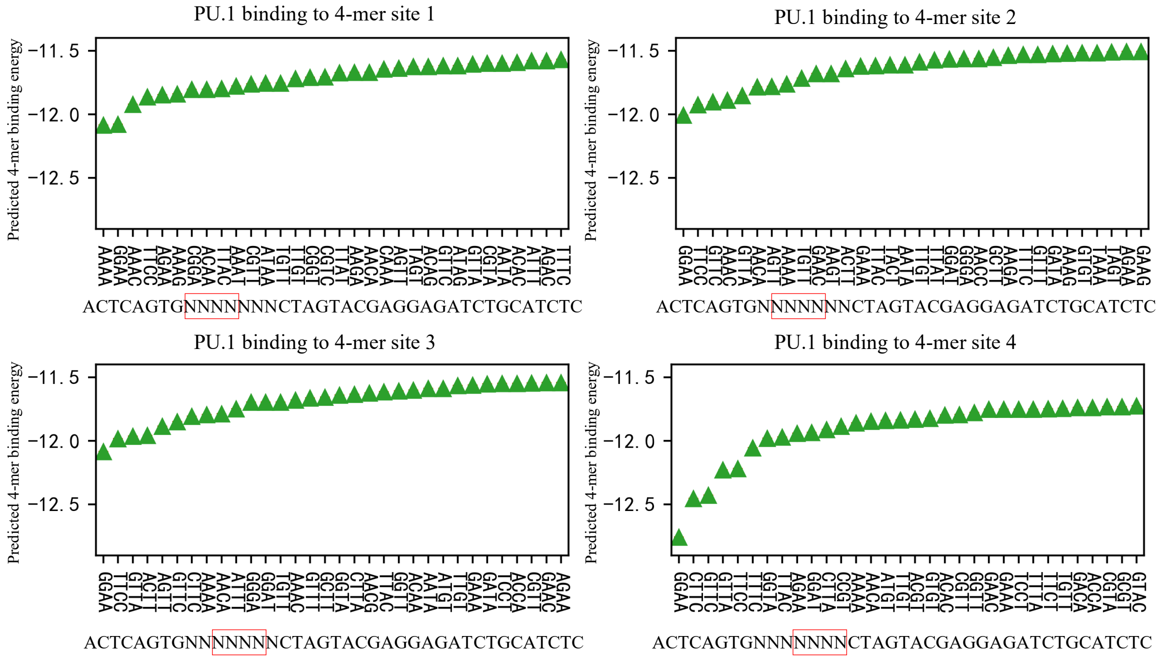

Figure S9. Plots of 4-mer binding energies at location 1, 2, 3 and 4 in the random region predicted by AEEscape for PU.1 DBD. The red box in the random region at the bottom of the figure indicates the location of the predicted binding energy landscape. The binding energies are the same as in Figure S8A. The 32 4-mer sequences with the lowest binding energy are shown.

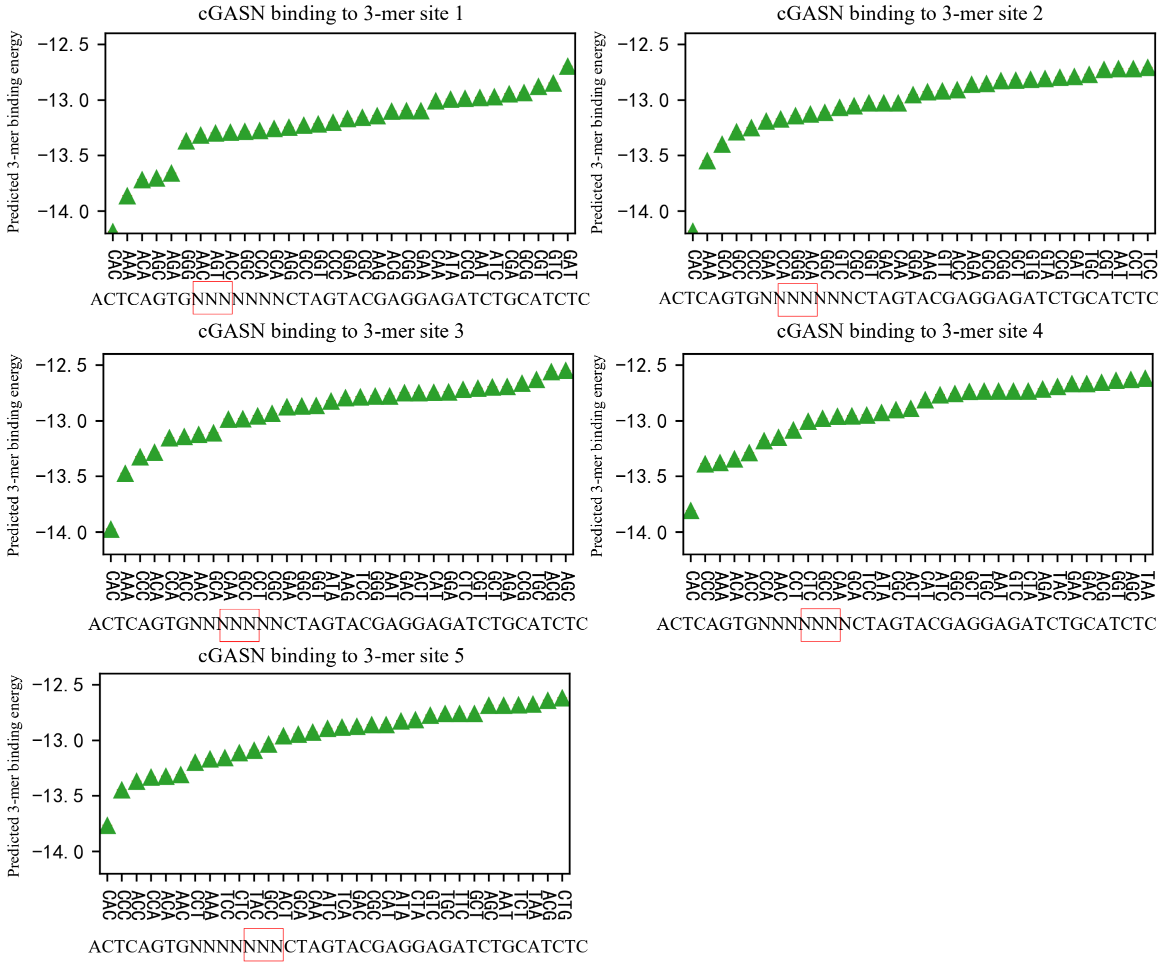

Figure S10. Plots of 3-mer binding energies at location 1, 2, 3, 4, and 5 in the random region predicted by AEEscape for cGASN. The red box in the random region at the bottom of the figure indicates the location of the predicted binding energy landscape. The binding energies are the same as in Figure S8B. The 32 3-mer sequences with the lowest binding energies are shown.

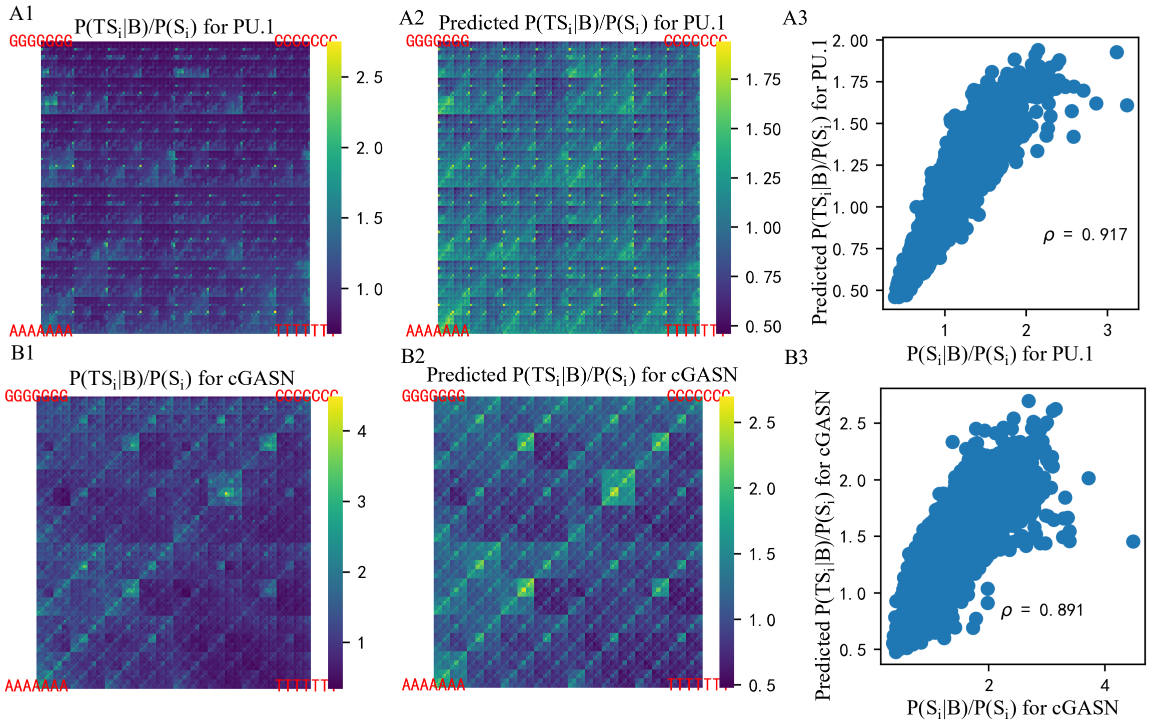

Figure S11. Comparative analysis of the probability ratios derived from KaScape experimental data and AEEscape predictions for PU.1 DBD and cGASN. (A1) The ratio of probabilities $P(TS_i|B)$ to $P(S_i)$ for PU.1 DBD, directly derived from KaScape experiments. (A2) Prediction ratios generated by the AEEscape algorithm, utilizing the same binding energy parameters as specified in Figure S8A. (A3) Comparison between the experimental data with the algorithmic predictions, with ρ denoting the Pearson correlation coefficient. (B1-B3) The same comparison analysis for cGASN. The AEEscape algorithm employs the identical binding energy parameters delineated in Figure S8B to facilitate the prediction.

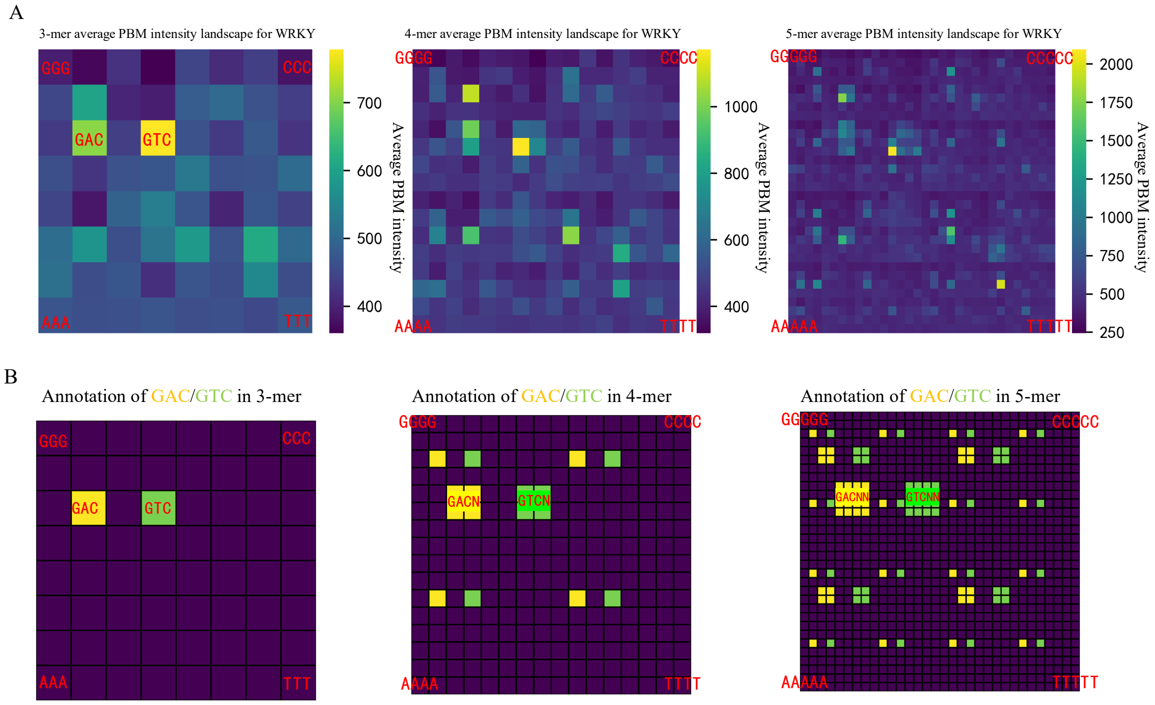

Figure S12. PBM intensity signals on K-mer graphs for *At*WRKY1, representing various sequence lengths derived from the same PBM data set. Data sourced from UniPROBE (accession UP00582), indicating median intensity for each 8-mer sequence. Intensity for shorter sequences is the mean of 8-mers encompassing the respective shorter sequence. (A) Average PBM intensity signals on 3-mer, 4-mer, and 5-mer graphs. (B) The positions containing GAC and GTC are marked in yellow and green respectively for 3-mer, 4-mer, and 5-mer graphs.

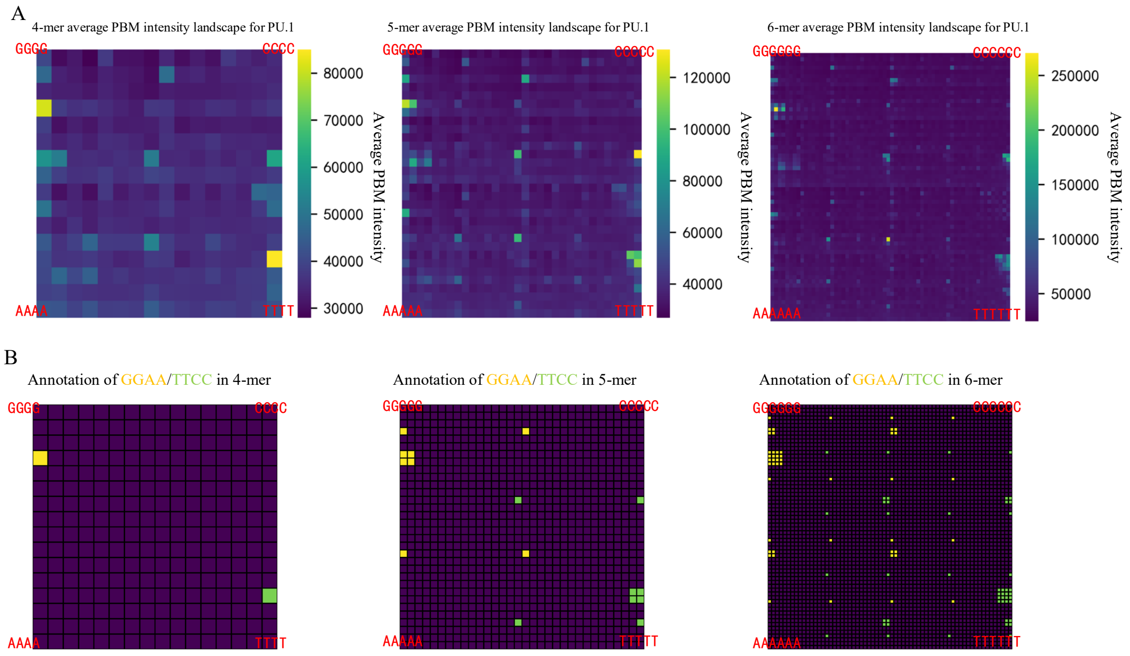

Figure S13. PBM intensity signals on K-mer graphs for Sfpi1 (PU.1), representing various sequence lengths derived from the same PBM data set. Data sourced from UniPROBE (accession UP00405), indicating median intensity for each 8-mer sequence. Intensity for shorter sequences is the mean of 8-mers encompassing the respective shorter sequence. (A) Average PBM intensity signals on 4-mer, 5-mer, and 6-mer graphs. (B) The positions containing GGAA and TTCC are marked in yellow and green respectively for 4-mer, 5-mer, and 6-mer graphs.

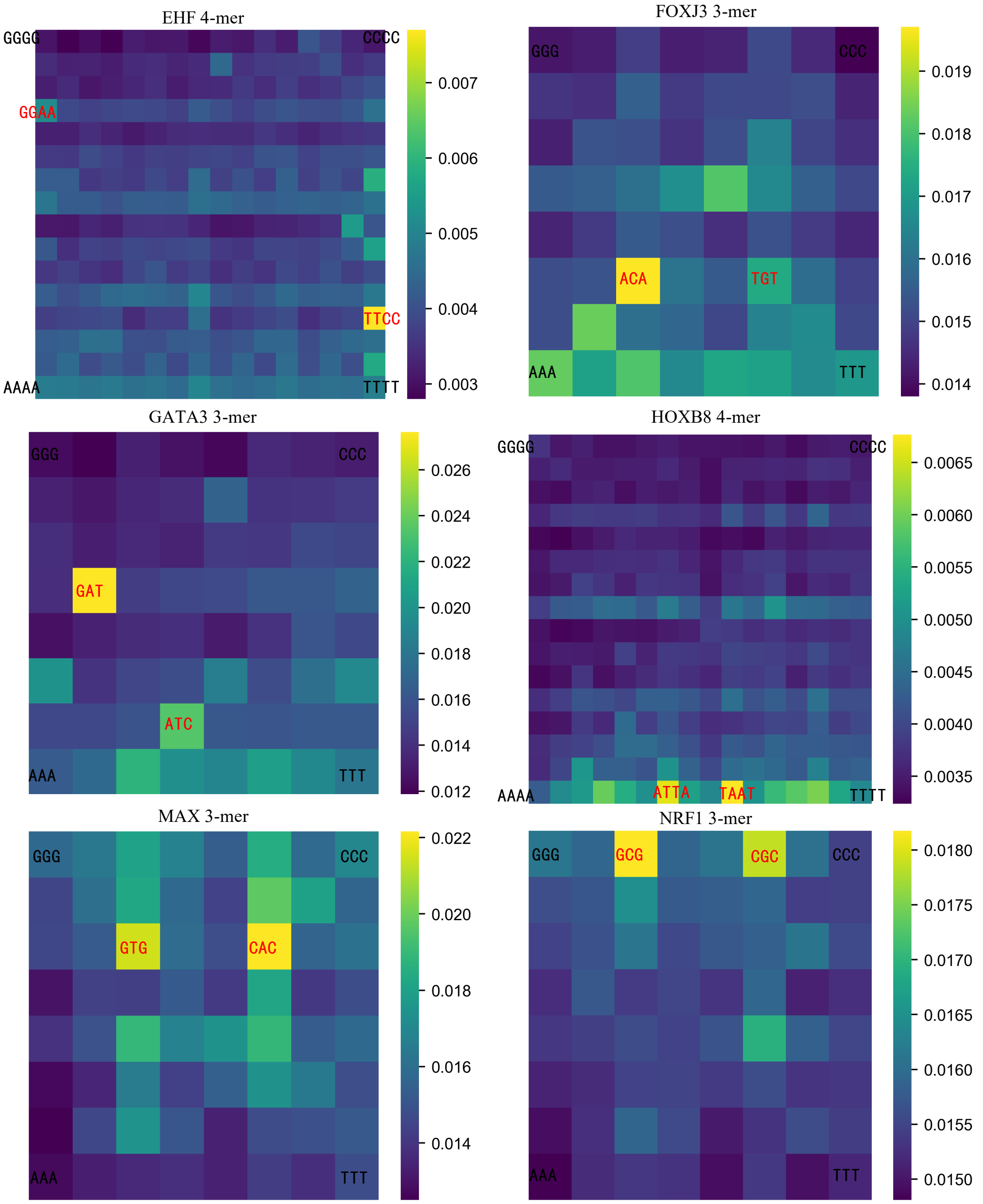

Figure S14. TF binding specificity on K-mer Graphs. PBM intensity signal distributions on K-mer graph are shown for the following transcription factors: EHF (ETS; UP00015), FOXJ3 (UP01497), GATA3 (UP00032), HOXB8 (UP00263), MAX (UP00060), and NRF1 (UP01608). The intensity distributions for k-mer sequences are derived from the normalized mean intensity of the debruijn sequences containing the respective k-mer, as obtained from

PBM data.[3] AEs are marked in red for each protein. Higher PBM intensity (approaching yellow) indicates stronger binding affinity, with AEs exhibiting the highest affinity.

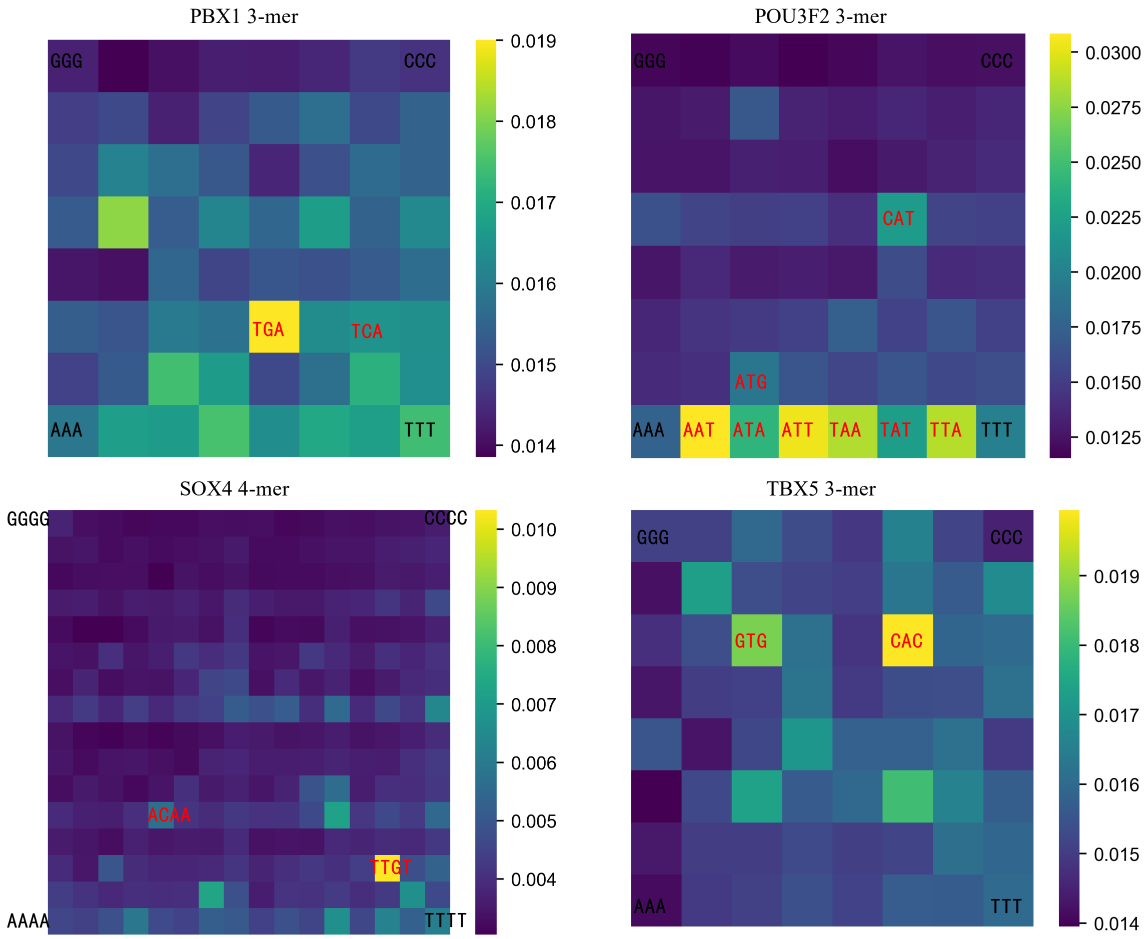

Figure S15. TF binding specificity on K-mer Graphs. The PBM intensity signal distributions on K-mer graph are shown for the following transcription factors: PBX1 (Homeobox; UniPROBE accession UP00185), POU3F2 (Homeobox, POU; UniPROBE accession UP00128), SOX4 (HMG_box; UniPROBE accession UP00062), and TBX5 (T-box; UniPROBE accession UP01398). The intensity distributions for k-mer sequences are derived from the normalized mean intensity of the debruijn sequences containing the respective k-mer, as obtained from PBM data.[3] AEs are marked in red for each protein. Higher PBM intensity (approaching yellow) indicates stronger binding affinity, with AEs exhibiting the highest affinity.

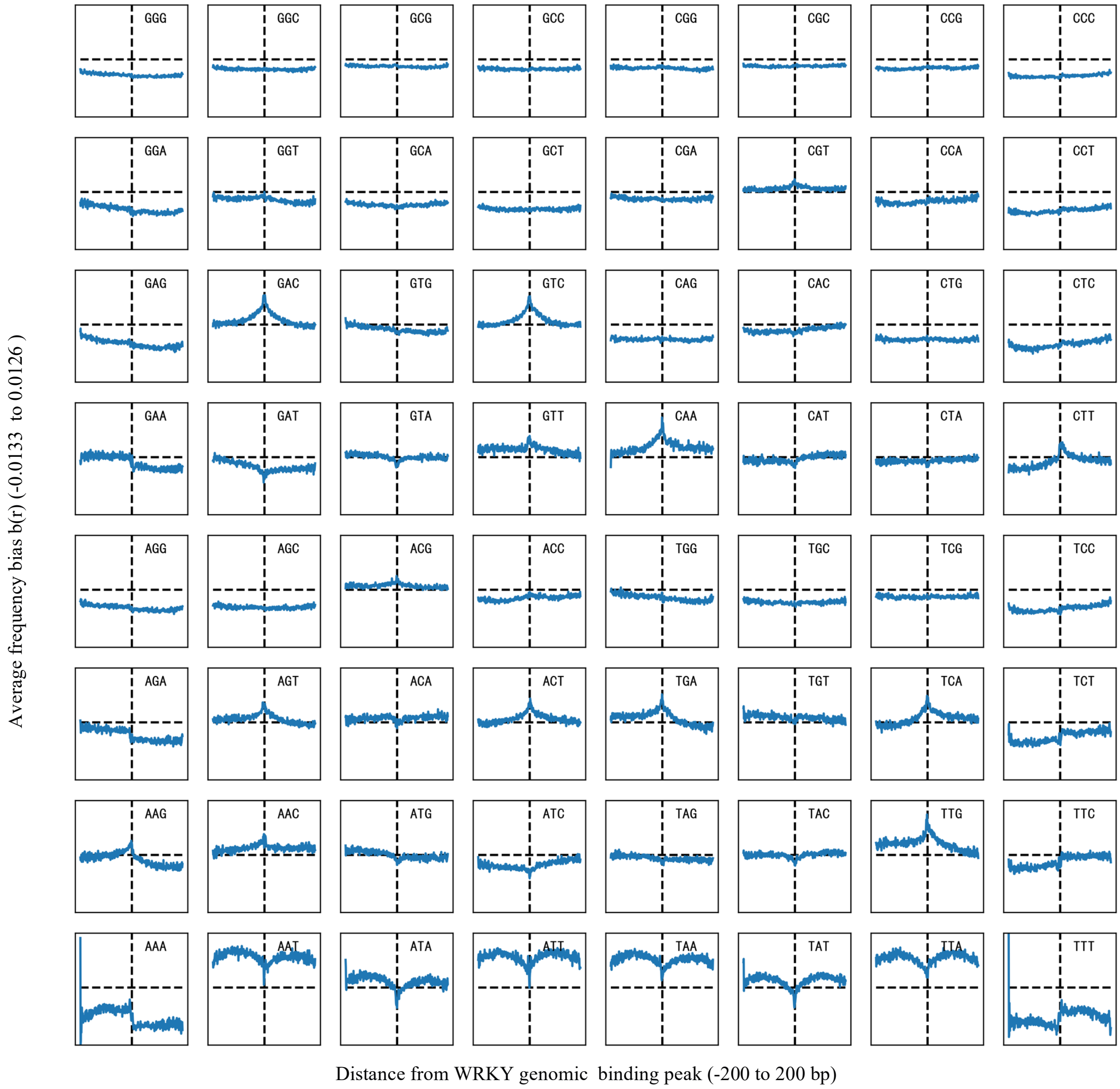

Figure S16. The average 3-mer frequency bias at WRKY genomic binding regions. All 3-mer average frequency biases plotted in a k-mer graph layout. Middle vertical line denotes x-axis value equal to 0 referred to as binding peak. Middle horizontal line represents y-axis value equal to 0.

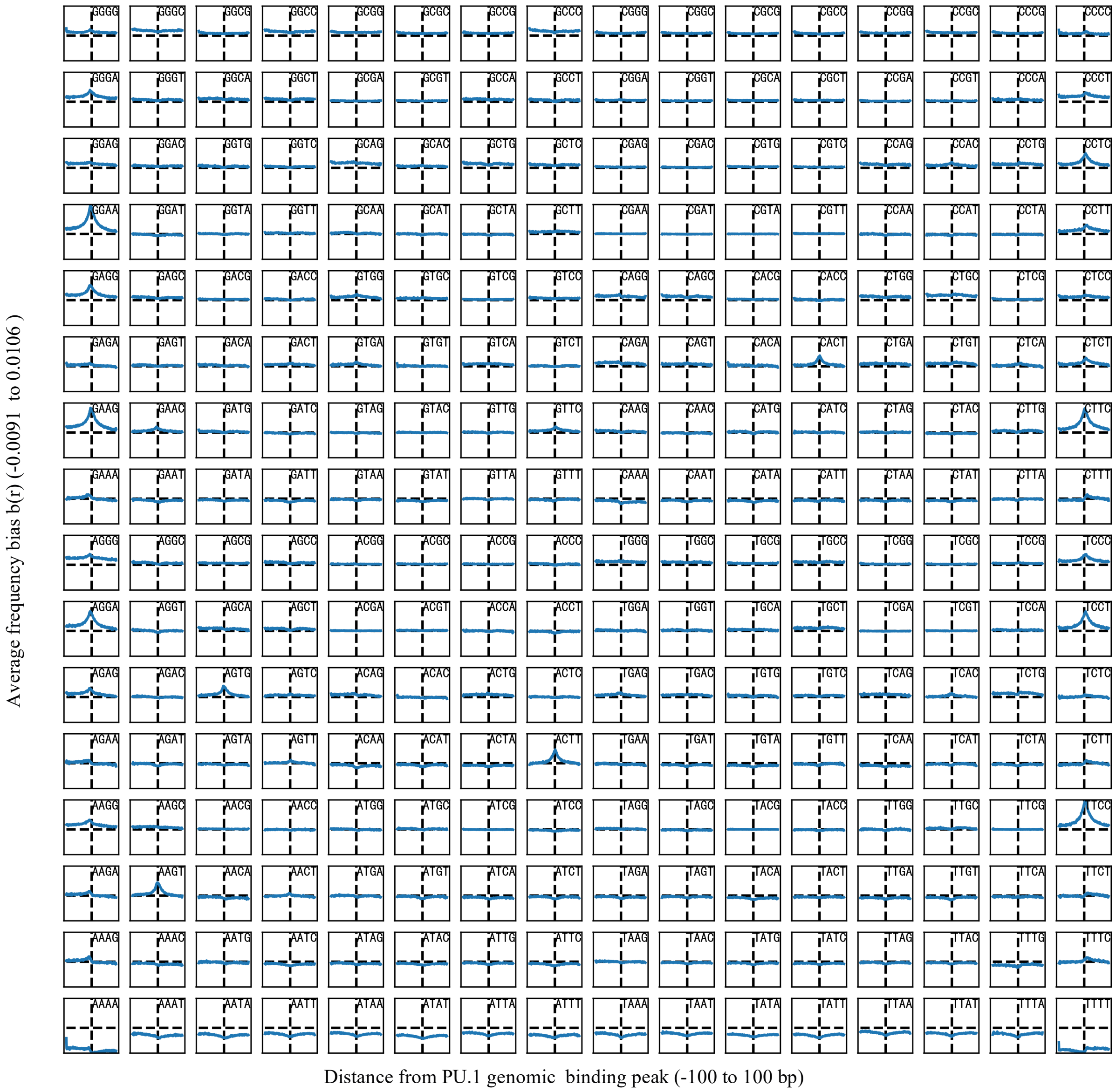

Figure S17. The average 4-mer frequency bias at PU.1 genomic binding regions. All 4-mer average frequency biases plotted in a k-mer graph layout. Middle vertical line denotes x-axis value equal to 0 referred to as binding peak. Middle horizontal line represents y-axis value equal to 0.

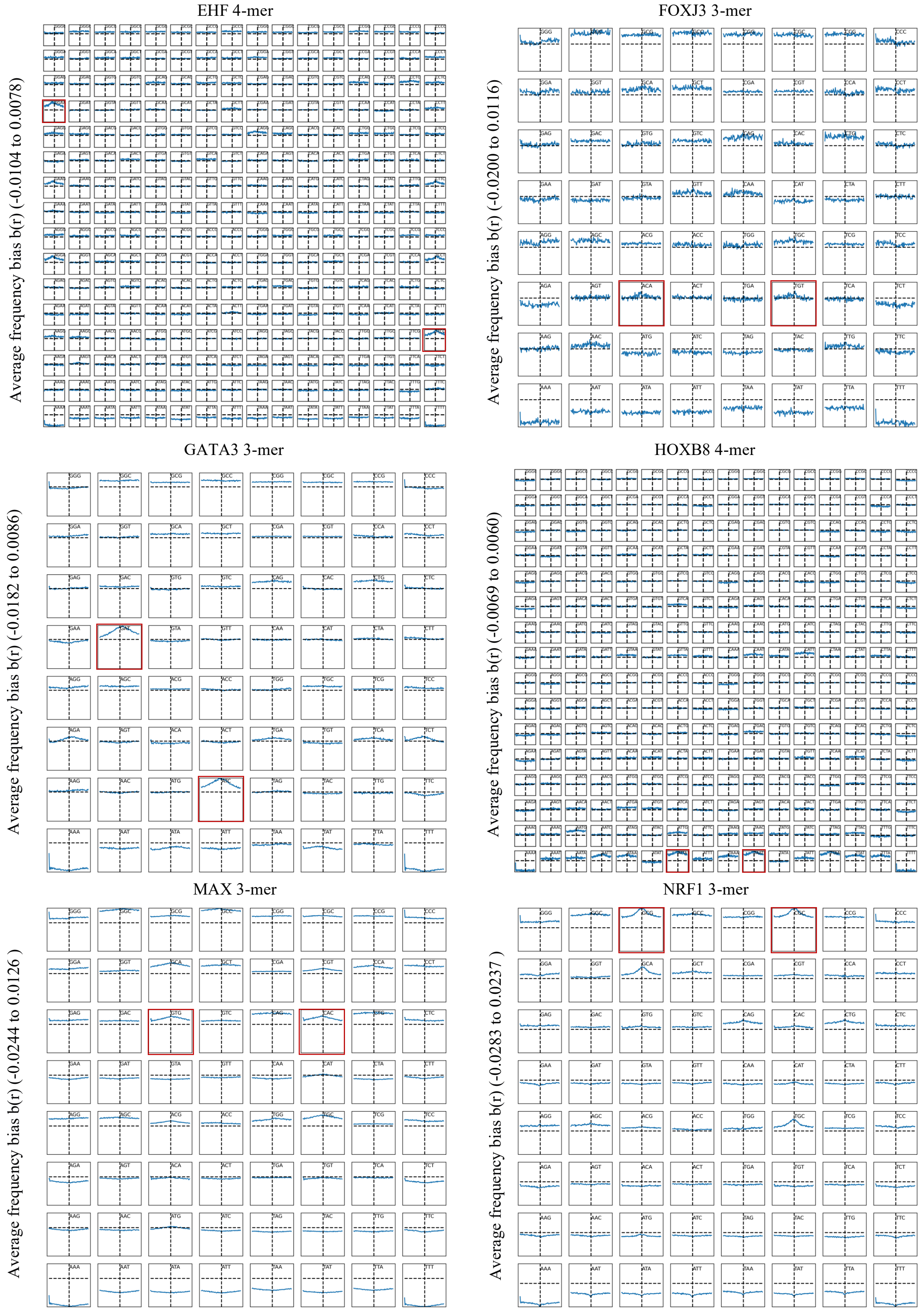

Figure S18. Average k-mer frequency bias at genomic binding regions for the transcription factors EHF, FOXJ3, GATA3, HOXB8, MAX, and NRF1. K-mer average frequency biases

are plotted in a k-mer graph layout, with the vertical line at x=0 corresponding to the binding peak annotated in the ReMap2022 dataset, and the horizontal line at y=0. Each curve represents a specific k-mer, and high-peak k-mers in the curves align with high-affinity k-mers in the heatmap (Figure S14), highlighting the functional relevance of AE density in the genomic regions.

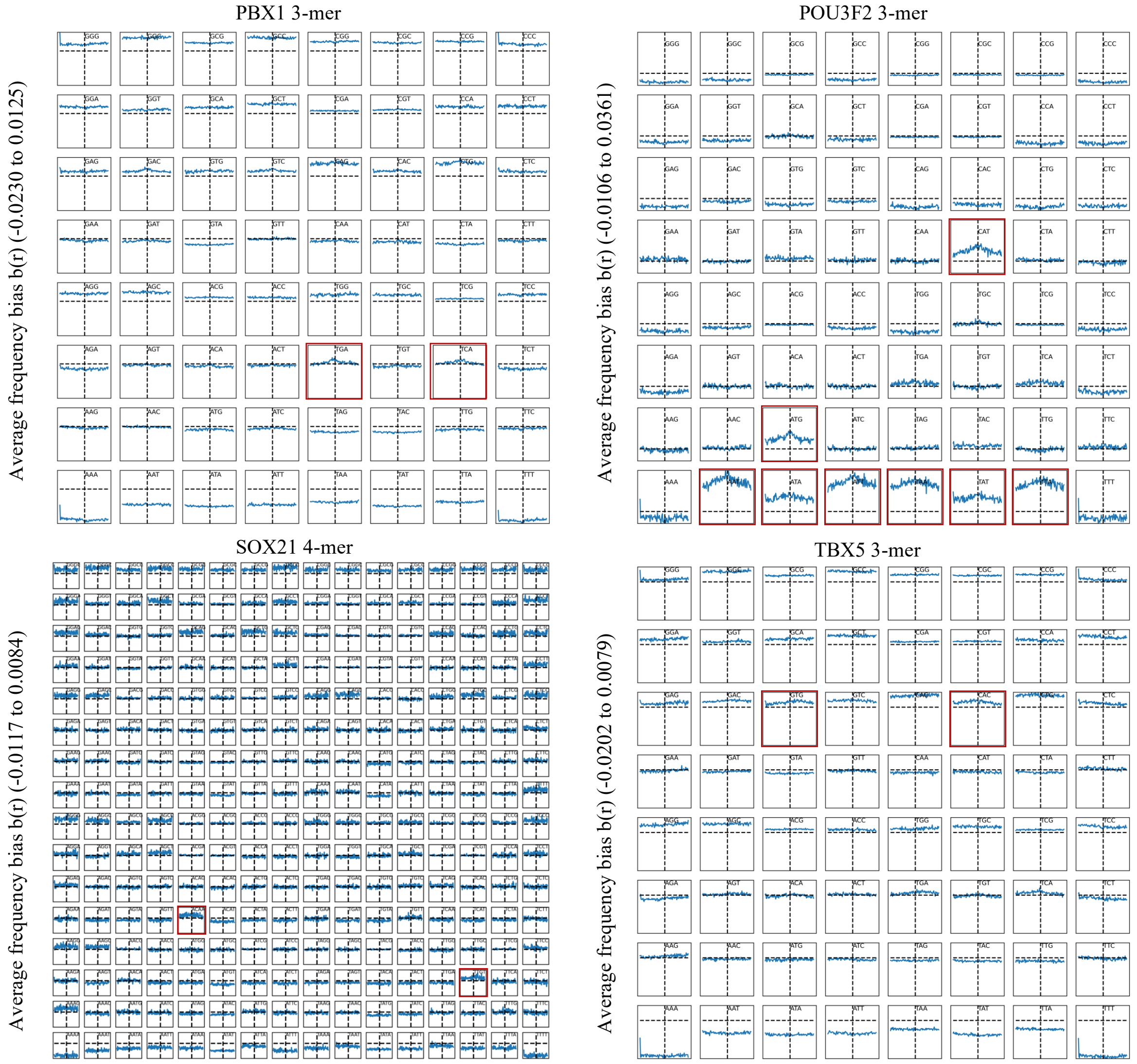

Figure S19. The average k-mer frequency bias at genomic binding regions for the transcription factors PBX1, POU3F2, SOX21, and TBX5. K-mer average frequency biases are plotted in a k-mer graph layout, with the vertical line at x=0 corresponding to the binding peak annotated in the ReMap2022 dataset, and the horizontal line at y=0. Each curve represents a specific k-mer, and high-peak k-mers in the curves align with high-affinity k-mers in the heatmap (Figure S15), highlighting the functional relevance of AE density in the genomic regions.

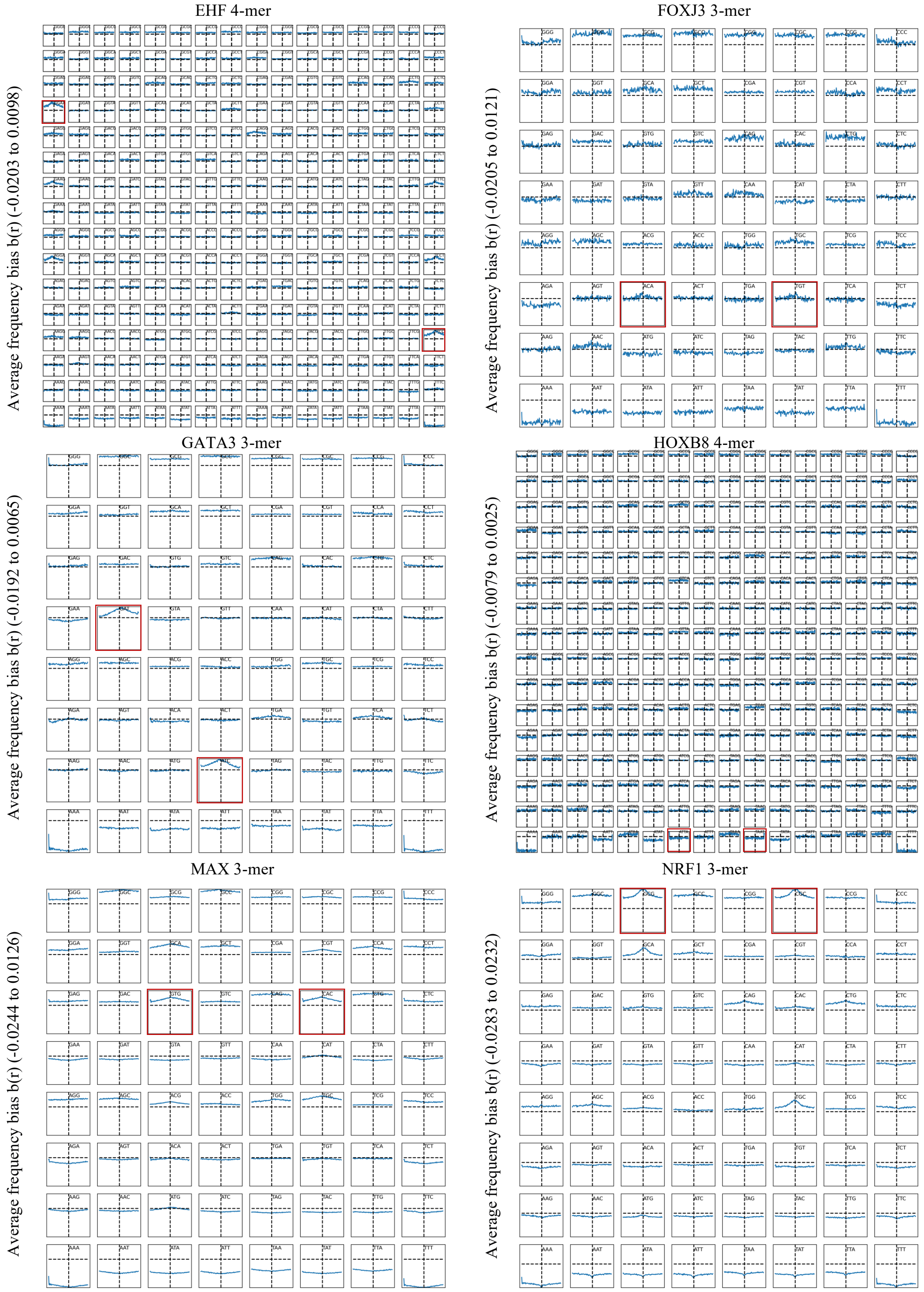

Figure S20. The average k-mer frequency bias at genomic binding regions for the transcription factors EHF, FOXJ3, GATA3, HOXB8, MAX, and NRF1. The sequences used for calculation exclude those containing the long consensus motif sequence. K-mer average frequency biases

are plotted in a k-mer graph layout, with the vertical line at x=0 corresponding to the binding peak annotated in the ReMap2022 dataset, and the horizontal line at y=0. Each curve represents a specific k-mer, and high-peak k-mers in the curves align with high-affinity k-mers in the heatmap (Figure S14), highlighting the functional relevance of AE density in the genomic regions.

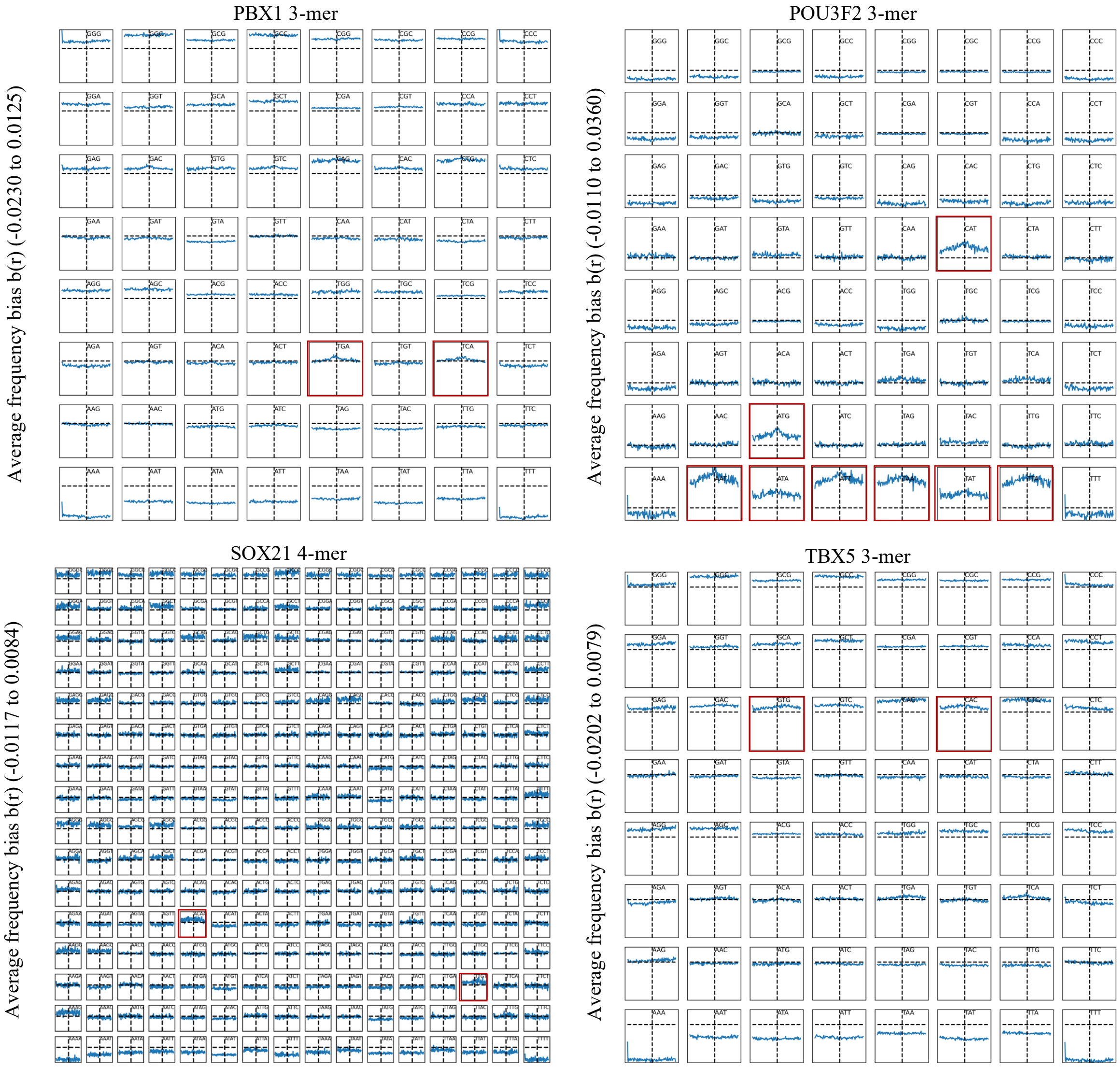

Figure S21. The average k-mer frequency bias at genomic binding regions for the transcription factors PBX1, POU3F2, SOX21, and TBX5. The sequences used for calculation exclude those containing the long consensus motif sequence. K-mer average frequency biases are plotted in a k-mer graph layout, with the vertical line at x=0 corresponding to the binding peak annotated in the ReMap2022 dataset, and the horizontal line at y=0. Each curve represents a specific k-mer, and high-peak k-mers in the curves align with high-affinity k-mers in the heatmap (Figure S15), highlighting the functional relevance of AE density in the genomic regions.

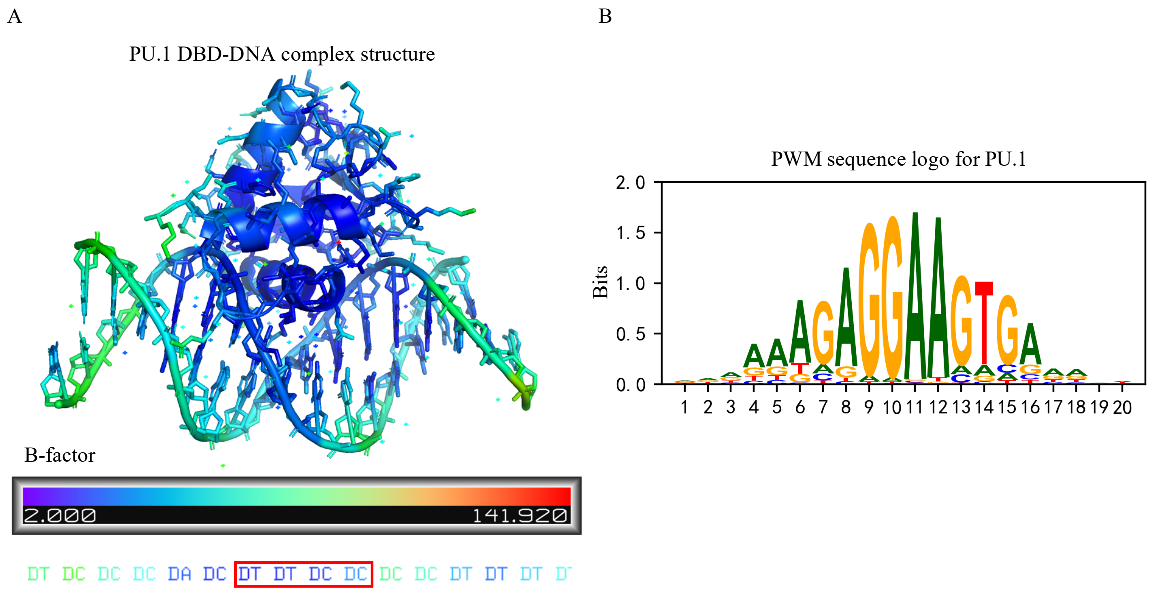

Figure S22. The structural and motif analysis of PU.1 DBD-DNA interaction. (A) Complex structure of PU.1 DBD with DNA (1pue.pdb). The structure is colored according to B-factor values, with bluer regions indicating lower B-factor and greater stability. The corresponding DNA sequence is displayed below, with the PU.1 covered region highlighted in a black box and the AE marked in a red box. (B) The PWM sequence logo from JASPAR database (MA0080.5) [3]

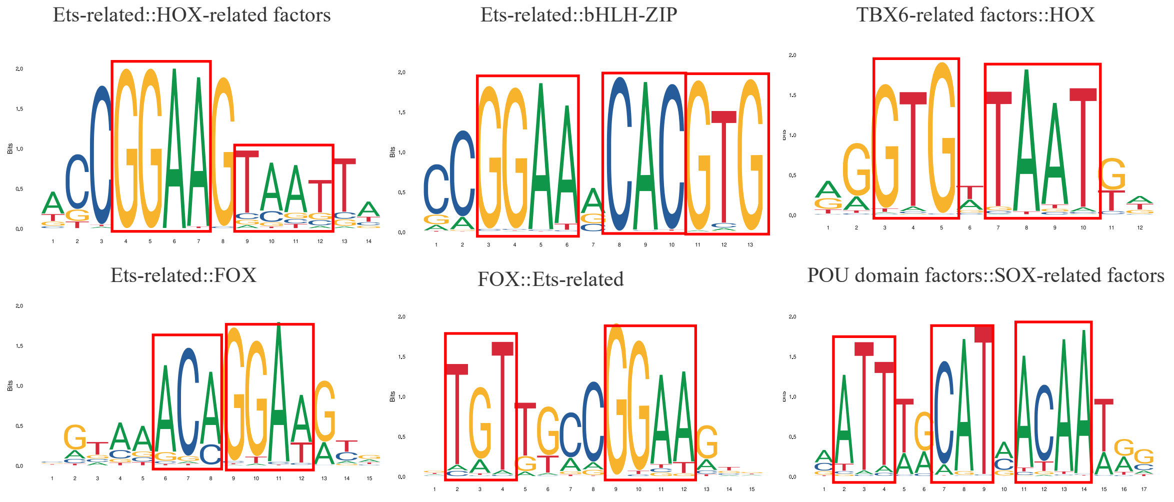

Figure S23. The PWM sequence logos for transcription factor combinatorial binding, sourced from the JASPAR database[4]. AEs, highlighted in red boxes, correspond to regions with the highest information content and are consistent with AEs derived from PBM data (Table S1). The combinatorial pairs are as follows: Ets-related::HOX-related factors include ELK1 and HOXA1 (MA1931.1); Ets-related::bHLH-ZIP factors include ERF and MAX (UN0507.2); TBX6-related factors::HOX include MGA and EVX1 (MA1960.1); Ets-related::FOX factors

include ETV2 and FOXI1 (MA1942.1); FOX::Ets-related factors include FOXO1 and ELF1 (UN0535.1); and POU domain factors::SOX-related factors include POU2F1 and SOX2 (MA1962.1).

Table S1 Transcription factors information from public data

Protein	Domain	AE	AE reverse complement	UniPROBE Number	PDB ID	peak number	peak number Exclude motif	Motif seed
EHF	ETS	TTCC	GGAA	UP00015	1PUE	47998	47979	ACCCGGAAGTA
FOXJ3	Fork_head	TGT	ACA	UP01497	2C6Y	15851	15030	RTAAACAA
GATA3	GATA	ATC	GAT	UP00032	3DFV	324537	250153	AGATAA
HOXB8	Homeobox	TAAT	ATTA	UP00263	1HDD	81024	47312	YMATTA
MAX	HLH	GTG	CAC	UP00060	1AN2	368364	368331	CACGTGNNNNNCACGTG
NRF1	unknown	GCG	CGC	UP01608	8K3D	90708	90116	YGCGCATGCGC
PBX1	Homeobox	TGA	TCA	UP00185	1LFU	63347	62882	ATCAATCAW
POU3f2	Homeobox, POU	AAT/ATG/ ATA/TAA	ATT/CAT/ TAT/TTA	UP00128	3D1N	10014	9631	NWTGMATAAWTNA
SOX4/ SOX21	HMG_box	TTGT	ACAA	UP00062	3U2B	4839	4839	AACAATNNNAKTGTT
TBX5	T-box	GTG	CAC	UP01398	2X6V	73692	73044	AGGTGTGA

Table S2 ReMap2022 data

Transcription factor	Peak number
WRKY	126424
PU.1	334755

Table S3 Proteins and random DNA types used in KaScape Experiments

Transcription factor	Random sequence type/Data set type
*At*WRKY1N	R1N3, R1N4 / input-bound dataset<br>R1N5 / unbound-bound dataset
PU.1 DBD	R2N4, R2N5, R2N6, R2N7 / input-bound dataset
cGASN	R2N5, R2N6, R2N7 / input-bound dataset

SI References

1. S. Ruan, S. J. Swamidass, G. D. Stormo, *Bioinformatics* **2017**, *33* (15), 2288.
2. H. Chen, Y. Xu, J. Jin, X.-d. Su, *Sci Rep.* **2023**, *13* (1), 16595.
3. M. A. Hume, L. A. Barrera, S. S. Gisselbrecht, M. L. Bulyk, *Nucleic Acids Res* **2015**, *43* (Database issue), D117.
4. I. Rauluseviciute, R. Riudavets-Puig, R. Blanc-Mathieu, J. A. Castro-Mondragon, K. Ferenc, V. Kumar, R. B. Lemma, J. Lucas, J. Chèneby, D. Baranasic, A. Khan, O. Fornes, S. Gundersen, M. Johansen, E. Hovig, B. Lenhard, A. Sandelin, W. W. Wasserman, F. Parcy, A. Mathelier, *Nucleic Acids Res.* 2024, 52 (D1), D174.
